# Hessian fly tolerance increases yield in elite durum wheat cultivars Svevo and Soft Svevo

**DOI:** 10.1038/s42003-026-10494-4

**Published:** 2026-06-15

**Authors:** Rachel D. Flynn, Devender Arora, Kumar Shrestha, Joe Louis, Subhash C. Minocha, Steven S. Xu, Subhashree Subramanyam

**Affiliations:** 1 Crop Production and Pest Control Research Unit, USDA-ARS, West Lafayette, IN USA; 2 Department of Entomology, Purdue University, West Lafayette, IN USA; 3 Bioinformatics Core, Purdue University, West Lafayette, IN USA; 4 Department of Entomology, University of Nebraska, Lincoln, NE USA; 5 Department of Biochemistry, University of Nebraska, Lincoln, NE USA; 6 Department of Biological Sciences, University of New Hampshire, Durham, NH USA; 7 Western Regional Research Center, USDA-ARS, Albany, CA USA

**Keywords:** Biotic, Herbivory

## Abstract

Insect resistance is critical for wheat crop sustainability and global food security. The dependence on innate plant resistance to prevent significant yield losses caused by Hessian fly (*Mayetiola destructor*), a major pest, could be detrimental in the event of severe infestation due to evolution of virulent biotypes. Wheat tolerance offers an alternate and viable strategy, as it minimizes selection pressure on pest populations with no plant yield penalty. Despite its lucrative attributes, the mechanisms governing tolerance are unknown. Here, we dissect Hessian fly tolerance-associated plant responses in two elite wheat (*Triticum turgidum* subsp. *durum*) cultivars, Svevo and Soft Svevo. Both exhibited dual phenotypes, harboring live and dead larvae, with phenotypic and molecular characteristics intermediate between resistant and susceptible plants. Although 75-96% of larvae survived on the infested tolerant plants, yields increased significantly by 14% over uninfested plants. Survival of the primary Hessian fly-infested stems was a key trait positively influencing yield (+31%) in the tolerant plants. The live larvae completed their life cycle producing viable adult Hessian flies. We further identified a positive correlation between caffeic acid levels and tolerance, suggesting a priming role in plant survival, associated with increased yield. Our finding provides the first comprehensive integrative physiological and metabolic characterization of Hessian fly tolerance advancing prospects for deploying tolerance with major implications for wheat productivity.

## Abstract (web summary)

Integrative phenotypic and molecular characterization reveals wheat tolerance to Hessian fly as a potential insect pest mitigation strategy for protecting crop productivity and promoting sustainable agriculture.

## Introduction

The Hessian fly (*Mayetiola destructor* Say), introduced into North America from Europe in the 18th century^1^, continues to be one of the most damaging insect pests, causing significant yield losses of wheat (*Triticum aestivum* L.) crops^2,3^. Newly hatched (neonates) 1st-instar Hessian fly larvae crawl to the base of the developing leaves in wheat seedlings, where they establish feeding sites. The larvae create shallow punctures on the leaf surface using their tiny mandibles and release salivary effectors into the plant cells. These secretions break down the cell wall barrier and reprogram the plant’s physiology to produce beneficial nutrients, leading to the creation of a nutrient sink and establishing susceptibility^4,5^. The feeding larvae undergo various developmental stages (1st-, 2nd-, 3rd-instar, and pupae), completing their development within 28–30 days. Resistance in wheat is governed by gene-for-gene interactions^6^. Based on this hypothesis, wheat exhibits resistance when attacked by Hessian fly expressing a cognate avirulence gene (incompatible interaction; dead avirulent larvae) but is susceptible when targeted by a Hessian fly lacking the cognate avirulence gene (compatible interaction; live virulent larvae). Resistant wheat plants maintain normal growth and yield, whereas the susceptible plants become stunted and often fail to survive, ultimately resulting in seedling death^6–8^.

For decades, the “go-to” environmentally-friendly and economical strategy used by wheat growers for managing and combating this insect pest has been to deploy resistant wheat cultivars harboring a dominant Hessian fly resistance (*R*) gene leading to larval antibiosis^9^. While 37 *R* genes (designated *H1*-*H36*, and *Hdic*) have been identified and mapped, only a few have been deployed in the field, largely based on their performance to provide robust resistance to diverse Hessian fly biotypes while maintaining grain yield^3,10^. Although highly effective, this strategy imposes enormous selection pressure on insect field populations, driving the emergence of virulent Hessian fly biotypes that may overcome native resistance in wheat in only 7 years^11,12^. Hence, relying heavily on this approach alone raises concerns about the validity of the strategy as a sustainable solution for maintaining wheat yields in the event of an outbreak of this insect pest.

An alternate, but less explored strategy that could complement native resistance is deploying tolerance to the Hessian fly. Tolerance is defined as the plant’s ability to maintain its fitness despite insect infestation through growth and reproduction^13,14^. Unlike in a susceptible plant, tolerance allows compensation, regrowth, and recovery under conditions of abundant pest infestation^15^. In wheat, tolerance is assumed to be associated with increased tillering post-insect infestation^16^. Additionally, unlike resistance, tolerance is believed to impose less selection pressure on the insect field populations, allowing most larvae feeding on tolerant plants to complete their development^13,14,17^. It is hypothesized that with tolerance, there is no penalty to either plant yield or insect development, circumventing the adaptive evolution of virulent fly populations in the field that may result in the breakdown of *R* genes.

Wheat tolerance to the Hessian fly has been variably described as “field-tolerance,” “field-resistance,” “partial-resistance,” and “partial-tolerance.” A few hexaploid wheat lines have been documented to exhibit tolerance to Hessian fly, including “Superb” (hard-red spring wheat), “Marquillo,” and “Massira.” These lines all show typical phenotypic characteristics of enhanced leaf growth, biomass, and tillers despite harboring at least one virulent Hessian fly larva^3^. However, the hypothesis that increased tillering is associated with tolerance^18^, as well as the genetic/molecular mechanisms underlying tolerance, remain unvalidated. A major Hessian fly field partial-resistance locus in a southern winter hexaploid wheat, “LA03136E71,” has been mapped^18^, while loci associated with the ability of Hessian fly-infested plants to prosper have been identified on chromosomes 1AL, 1AS, and 3DL^19^. Resistance in the hexaploid Marquillo is believed to be a form of Hessian fly tolerance with recessive gene expression and suggested to be associated with increased tillering post-infestation^20^.

Wheat tolerance to Hessian fly has received limited attention^16^ and is underutilized primarily due to (i) challenges in identifying the tolerance trait; (ii) difficulty in measuring plant yield; (iii) lack of tolerance-associated molecular markers; and (iv) a critical knowledge gap in the genetic and molecular mechanisms underlying this trait^14^. Detailed characterization of this phenomenon is key to the effective implementation of this strategy for mitigating pest damage and significant losses to wheat yield. In the current study, we characterize the phenotypic and molecular responses of two elite tetraploid durum wheat (*T*. *turgidum* subsp. *durum*) cultivars, “Svevo” and “Soft Svevo,” that we identified as tolerant to Hessian fly infestation. We provide detailed insights into the responses observed during Hessian fly tolerance, offering a valuable resource for developing and implementing tolerance as an insect mitigation strategy to complement native resistance, with major implications on sustainable wheat yield.

## Results

### Tolerant Svevo shows dual phenotypic responses to Hessian fly larval feeding

**Fig. 1:**
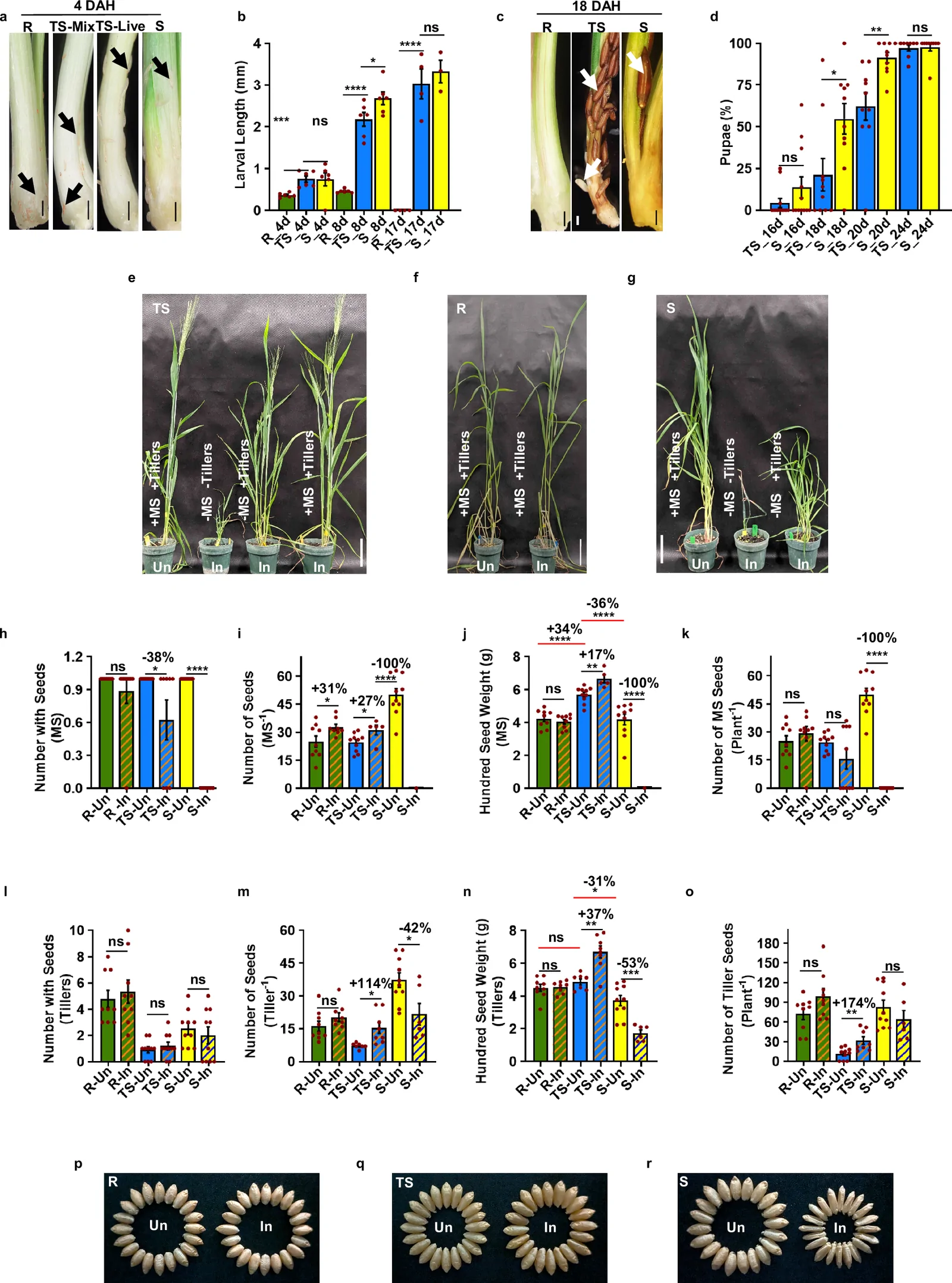
Phenotypic responses of durum wheat to Hessian fly larval feeding. **a** Representative photomicrograph of dead (red, 1st instar) larvae on PI 519832 (resistant, R), mix of dead and live (white, 2nd instar) larvae on Svevo (tolerant, TS-Mix), live (2nd instar) larvae on Svevo (tolerant, TS-Live), and live (2nd instar) larvae on PI 61188 (susceptible, S) infested durum wheat plants at 4 DAH (days after egg hatch). Black arrows show larvae. Scale bars, 1 mm. **b** Comparison of larval length in R, TS, and S plants at 4, 8, and 17 DAH (d). **c** Representative photomicrographs lacking larvae on R, live (white, 3rd instar) larvae and pupae on TS, pupae on S infested durum wheat plants at 18 DAH. White arrows show larvae and pupae. **d** Comparison of percentage (%) pupae in TS and S plants at 16, 18, 20, and 24 DAH (d). **e**–**g** Representative pot photos of uninfested (Un) and infested (In) R, TS, and S genotypes showing variable phenotypes with (indicated by “+”) or without (indicated by “−”) MS (main stem; primary Hessian fly-infested stem) and tillers. Scale bars, 9 cm. Yield data were taken from uninfested (Un) and infested (In) R, TS, and S plants for **h** number of MS with seeds. **i** Number of seeds per MS. **j** Hundred seed weight (g) from MS. **k** Number of seeds produced by MS per plant. **l** Number of tillers with seeds. **m** Number of seeds per tiller. **n** Hundred seed weight (g) from tillers. **o** Number of seeds produced by tillers per plant. **p**–**r** Representative photos of seeds harvested from uninfested (Un) and infested (In) R, TS, and S plants. Scale bars, 0.5 cm. Statistical analysis (unpaired two-tailed t-test) was performed with GraphPad Prism (v10). Lines above the bars represent significant comparisons between R, TS, and S: black lines indicate Un vs. In within a genotype; red lines indicate Un vs Un between genotypes. The number (*n*) of replicates for each experiment are provided in Supplementary Data 3. Error bar represents the standard error of mean. *p ≤ 0.05, **p ≤ 0.01, ***p ≤ 0.001, and ****p ≤ 0.0001.

To assess the phenotypic responses of the tolerant Svevo (designated as TS) cultivar compared to the resistant (PI 519832^21^; designated as R) and susceptible (PI 61188^22^; designated as S) wheat, plants were infested with Hessian fly biotype L. As expected, the biotype L Hessian fly-infested R and S plants were homozygous, with R plants (100%) harboring only dead, red, 1st instars, and S plants (90%) having live, white, 2nd instars by 4 days after egg hatch (DAH) (Fig. 1a and Table 1). In contrast, infested TS plants showed two phenotypic responses to biotype L, with 50% (*n* = 33/66) harboring both live and dead larvae (TS-Mix) and 50% (*n* = 33/66) harboring only live larvae (TS-Live) by 4 DAH (Fig. 1a and Table 1). Similar dual (TS-Mix and TS-Live) phenotypic responses, ranging from 30 to 77% of TS-Mix, were also observed in TS plants infested with other Hessian fly biotypes, including C, B, O, D, and *vH13* (Table 1). Compared to the S plants, live larvae on the TS plants were significantly smaller at 8 DAH, but by 17 DAH had attained the same length (*p* = 0.5606) (Fig. 1b). By 18 DAH, less than 20% of the larvae on the TS plants had pupated while others remained as 3rd instars (Fig. 1c, d). However, by 24 DAH, all live larvae on the TS plants were pupated, resembling the S plants (Fig. 1d). At 22 DAH, 100% of the biotype L-infested R plants showed normal growth having 1–5 ligules (Supplementary Fig. 1a) and 4–7 leaves (Supplementary Fig. 1b) with no significant difference in length compared to the uninfested plants. In contrast, 50% of infested TS plants showed stunted growth with significantly (*p* ≤ 0.05) smaller ligules 3–5 and leaves 4–7 (Supplementary Fig. 1c, d). The remaining 50% of TS plants showed all ligules and leaves 3–5 comparable in growth to the uninfested plants (Supplementary Fig. 1c, d). The leaves 6–7 in the TS plants showed mild stunting compared to the uninfested controls (Supplementary Fig. 1d). All the S plants showed a stunted growth and were missing ligules 3–5 (Supplementary Fig. 1e) and leaves 4–6 in most plants, with leaf 3 being significantly stunted (*p* = 0.0002) compared to the uninfested plants (Supplementary Fig. 1f).

**Table 1:** Phenotypic responses of Svevo to Hessian fly

Biotype	# plants	Avg. larvae	# plants (#LL)	# plants (#DL)	# plants mixed (#LL: #DL)	% plants (mixed response)	% DL
Response of TS (Svevo) plants							
L	66	15	33 (479)	0	33 (382:130)	50	13
C	11	14	4 (37)	0	7 (108:11)	63	8
B	10	7	7 (40)	0	3 (27:4)	30	6
O	10	7	4 (19)	1 (1)	5 (34:12)	50	20
D	9	15	2 (21)	0	7 (96:36)	77	24
vH13	20	12	13 (47)	1 (2)	6 (83:10)	30	5
Response of R (PI 519832) plants^a^							
L	16	13	0	16 (207)	0	0	100
Response of S (PI 61188) plants^b^							
S	17	13	16 (196)	0	0	0	0

# plants: total number of plants evaluated.

Avg. larvae: average number of larvae per plant.

# plants (#LL): number of plants harboring only live larvae (LL). The total number of LL from all plants is given in parentheses.

# plants (#DL): number of plants harboring only dead larvae (DL). The total number of DL from all plants is given in parentheses.

# plants mixed (#LL:#DL): number of plants harboring both LL and DL. The total numbers of LL and DL from all plants are given in parentheses.

% plants (mixed response): percentage of plants showing mixed response harboring both LL and DL.

% DL: percentage of DL in all plants.

^a^Hessian fly resistant wheat control.

^b^Hessian fly susceptible wheat control.

Infested TS plants showed variability in plant phenotype with respect to growth and yield (Fig. 1e). These include (i) 50% (6/12) plants with both the primary Hessian fly-infested stem (designated as main stem; MS) and tillers surviving and producing seeds (+MS +Tillers); (ii) 33% (4/12) plants with the MS dead and only the tillers producing seeds (−MS +Tillers); and (iii) 17% (2/12) plants with both MS and tillers dead and neither producing seeds (−MS −Tillers) (Fig. 1e). In contrast, the R plants clearly showed only one phenotype with 100% (9/9) plants surviving and both MS and tillers producing seeds (Fig. 1f). The S plants showed two phenotypes with 60% (9/15) plants with MS dead and only tillers producing seeds, while the remaining 40% (6/15) with both MS and tillers dead with neither producing seeds (Fig. 1g). We found most TS plants that showed MS with seeds had up to 6 leaves, while the MS of TS plants with stunted ligules and leaves did not produce seeds. Interestingly, all infested TS plants except the 17% that produced no seeds, including the ones with stunted ligule, leaves, and −MS (Supplementary Fig. 1b, c), produced tillers with seeds, and yields comparable to the uninfested plants, with some having increased seed number and hundred seed weight (Fig. 1h–o). The rapid regrowth and higher yield in the TS plants following Hessian fly infestation clearly suggests involvement of compensatory growth as a tolerance mechanism contributing to plant yield.

### Plant yield in Hessian fly-infested Svevo increases with few penalties to larval development

We next carried out a detailed analysis on the impact of Hessian fly infestation on plant yield in TS plants. Despite the infested TS plants showing a reduction (−38%; *p* = 0.034) in the number of MS with seeds (Fig. 1h), there was a significant increase (unpaired two-tailed t-test) in the (i) average number of seeds per MS (+27%; *p* = 0.034) that survived, and (ii) seed weight (+17%; *p* = 0.003) compared to the uninfested plants (Fig. 1i, j). Additionally, the number of seeds from MS per plant in TS infested plants were comparable to the uninfested controls, resembling the response observed in R wheat (Fig. 1k). Unlike the R and TS plants, since none of the S plants showed MS survival, the MS yield was reduced by 100% compared to the uninfested plants (Fig. 1h–k). Unlike the MS, the infested plants from all three genotypes (R, TS, and S) had tillers producing seeds that were comparable to the uninfested plants (Fig. 1l). Notably, the infested TS plants showed significantly increased average seed number per tiller and weight by +114% (*p* = 0.0112) and +37% (*p* = 0.0011), respectively, unlike the R plants that remained unchanged for these traits compared to the uninfested plants (Fig. 1m, n). Surprisingly, the seed weight from MS in uninfested TS plants was 34% (*p* = 0.0001) and 36% (*p* = 0.0006) higher compared to the uninfested R and S plants, respectively (Fig. 1j), and 31% (*p* = 0.0213) higher than the uninfested S plants from tillers (Fig. 1n). Although the number of tillers with seeds in infested S plants were comparable to the uninfested controls, there was a significant drop in both the average seed number per tiller (−42%, *p* = 0.0159) (Fig. 1m) and weight (−53%, *p* = 0.0004) (Fig. 1n). This resulted in a decrease in total number of seeds produced by the tillers (−54%, *p* = 0.01) in S plants. In contrast, TS plants showed comparable seed numbers produced by MS but dramatically increased numbers (+174%, *p* = 0.004) produced by the tillers, while R plants had numbers similar to the uninfested controls (Fig. 1k, o). Representative photographs of harvested seeds from infested R (Fig. 1p) and TS (Fig. 1q) plants clearly showed seed quality comparable to those from uninfested plants, while the seeds from the infested S plants were small and shriveled (Fig. 1r). Thus, there is a distinct difference in the quality of tillers arising in S plants compared to TS plants. The increased tillering contributing to yield in TS plants is directly related to tolerance. Although around 13% of biotype L larvae died on the TS plants (Table 1), the remaining live larvae successfully pupated and emerged as adults. The adult flies that emerged from TS plants were viable and successfully infested susceptible (“Newton”) hexaploid wheat plants, laid eggs, and completed their life cycle. These data clearly demonstrate that yield increases significantly in TS plants, with minimal penalty to the larvae.

### Main stem survival trait selection increases Svevo Hessian fly tolerance and plant yield

**Fig. 2:**
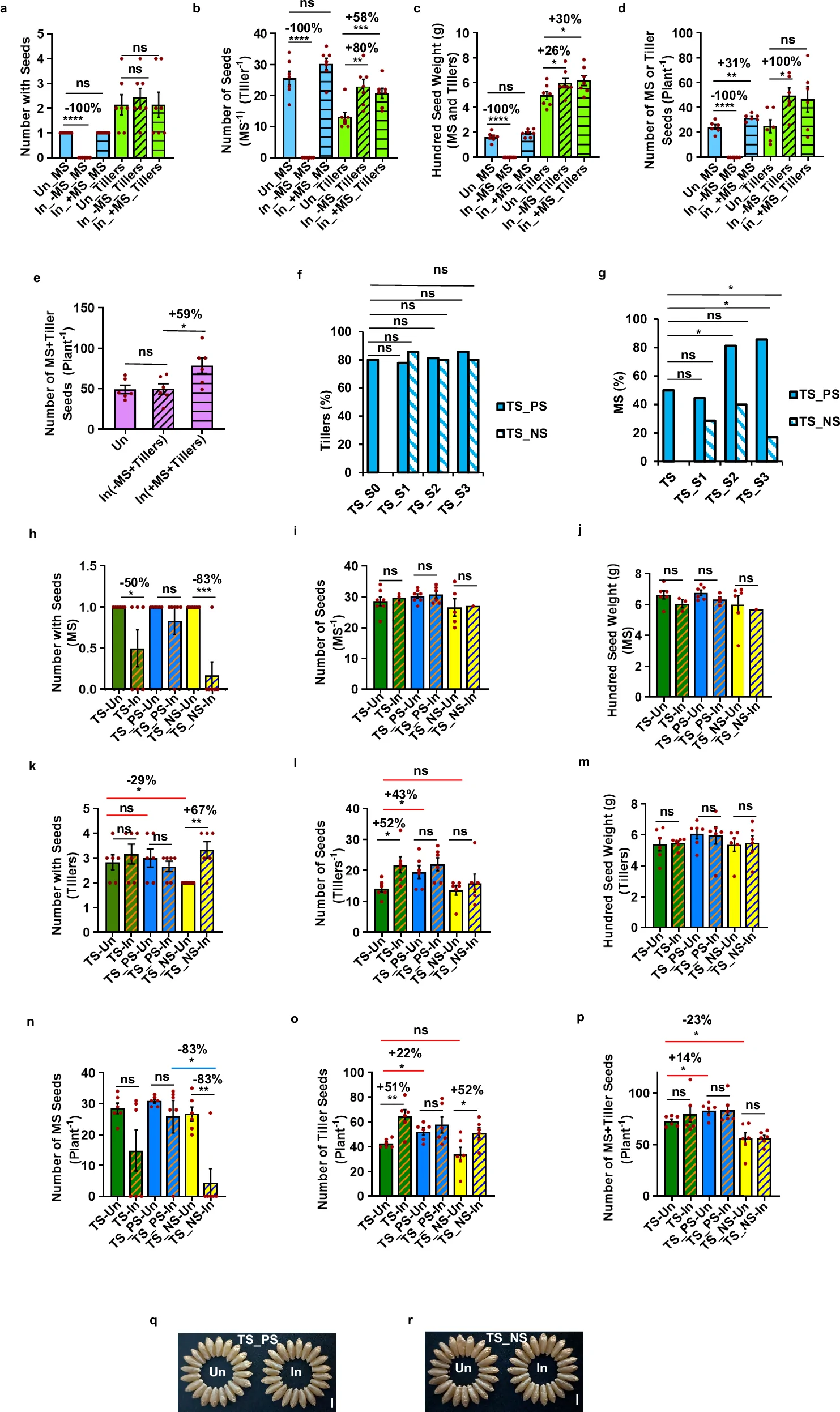
Selection for main stem survival trait in Hessian fly-infested Svevo wheat. Yield data was taken from uninfested (Un) and biotype L-infested (In) unselected Svevo (TS, tolerant) main stems (MS; primary Hessian-fly infested stem) and tillers in plants with (+MS) and without (−MS) MS that produced seeds for **a** number of MS and tillers with seeds. **b** Number of seeds per MS and tiller. **c** Hundred seed weight (g) from MS and tillers. **d** Number of MS or tiller seeds produced per plant. **e** Number of MS+tiller seeds produced per plant. Percentage (%) of plants producing **f** tillers and **g** MS with seed post-infestation through three generations of selfed (“S”) selection in TS_PS (positively selected for MS survival trait) and TS_NS (negatively selected for MS survival trait) plants. S0-S3 represents the number of selfed generations. **h**–**m** Yield data taken from uninfested (Un) and infested (In) TS, TS_PS, and TS_NS plants from S_3_ generation for **h** number of MS producing seeds. **i** Number of seeds per MS. **j** Hundred seed weight (g) from MS. **k** Number of tillers producing seeds. **l** Number of seeds per tiller. **m** Hundred seed weight (g) from tillers. **n** Number of MS seeds per plant. **o** Number of tillers seeds per plant. **p** Number of seeds (MS+tillers combined) per plant. Error bars represent standard error of the mean. Significance bars represent comparisons between uninfested (Un) and infested (In) unselected (TS) and selected lines (TS_PS, TS_NS); black indicates Un vs In within the same line; red indicates Un vs Un between unselected and selected lines; blue indicates In vs In between selected lines. **q**, **r** Representative photos of seeds harvested from uninfested (Un) and infested (In) TS_PS and TS_NS plants. Scale bars, 0.5 cm. Statistical analysis (unpaired two-tailed t-test) was performed with GraphPad Prism (v10). The number (*n*) of replicates for each experiment are provided in Supplementary Data 3. *p ≤ 0.05, **p ≤ 0.01, ***p ≤ 0.001, and ****p ≤ 0.0001.

To determine if survival of the MS (primary Hessian fly-infested stem) influences plant yield in TS plants, we collected and compared the yield data (unpaired two-tailed t-test) between TS plants exhibiting the main stem survival trait (+MS) and plants lacking the main stem (−MS) (Fig. 2a–e). In the +MS plants, there was no significant difference in the number of MS with seeds, average seed number per MS, and weight compared to the uninfested plants (Fig. 2a–c). However, a 31% (*p* = 0.008) increase in average seed number from MS per plant was observed in the +MS infested plants (Fig. 2d). As expected, in the TS plants lacking MS (−MS), there was 100% decrease for all the traits associated with MS (Fig. 2a–d). While correlation coefficient analysis revealed lack of significant difference (*r* = 0.3948; *p* = 0.4385) in the number of tillers producing seeds from both the −MS and +MS we observed a significant increase in seed number per tiller and seed weight compared to the uninfested controls (Fig. 2a–c). Interestingly, the number of seeds produced by tillers per plant doubled in the −MS plants (Fig. 2d), with an overall increase of seeds per plant (+59%; *p* = 0.021) in the +MS plants (Fig. 2e). These data unambiguously demonstrate that TS plants displaying the MS survival trait have increased yields.

Since the infested TS wheat had an overall higher yield (from MS+tillers) in plants exhibiting the MS survival trait (+MS) compared to the infested TS plants lacking the main stem (−MS), we hypothesized that MS survival is key for the increased yield in the tolerant wheat lines. To validate this hypothesis, we collected data using the following two selection parameters: (i) positive selection (PS) for MS survival trait (+MS); and (ii) negative selection (NS) for loss of MS survival trait (−MS) in TS plants, for four generations (Supplementary Fig. 2). While there was no difference in the percentage of plants with tillers that were either positively selected for (TS_PS) or negatively selected against (TS_NS) the MS survival trait (Fig. 2f), we observed a significant increase in percentage of MS survival by the 4th generation in the TS_PS plants (86%; *p* = 0.03) and a significant decrease in the TS_NS plants (20%; *p* = 0.02) compared to the unselected plants (Fig. 2g). These data reveal that selection for or against the MS survival fine-tunes the genetic control of tolerance.

With respect to yield from MS following selection, there was a significant decrease in the number of MS with seeds in the infested TS_NS plants (−83%) and the unselected TS plants (−50%) that was not observed in the TS_PS plants (Fig. 2h). None of the other traits were significantly different between the selected and unselected or the infested and uninfested plants (Fig. 2i, j). With respect to tillering, the infested TS_NS plants showed an increase compared to the uninfested controls (Fig. 2k). Notably, there was a decrease in number of tillers produced in uninfested TS_NS plants compared to the uninfested TS unselected and uninfested TS_PS plants (Fig. 2k). Interestingly, the average seed number per tiller increased by 43% in the uninfested TS_PS plants compared to the unselected uninfested plants (Fig. 2l). No change was observed in the seed weight for all the lines (Fig. 2m). The number of seeds per plant produced by infested MS decreased (−83%, *p* < 0.01) in the TS_NS plants compared to the infested TS_PS and uninfested TS_NS plants (Fig. 2n). While the total seeds produced from tillers in the infested TS_NS and TS unselected plants increased by around 50% compared to the uninfested controls, we also observed a 22% increase in the uninfested TS_PS plants compared to the unselected, uninfested plants (Fig. 2o). The total seed numbers produced by MS and tillers increased by 14% in the uninfested TS_PS plants and decreased by 21% in the TS_NS plants compared with the uninfested, unselected TS plants. However, there was no significant difference in the number of seeds between uninfested and infested plants for all three lines (Fig. 2p). Our data clearly demonstrates that selecting the TS plants for the MS survival trait significantly increased the overall yield in both the infested and uninfested plants.

### Hessian fly infestation induces early defense gene expression in Svevo

**Fig. 3:**
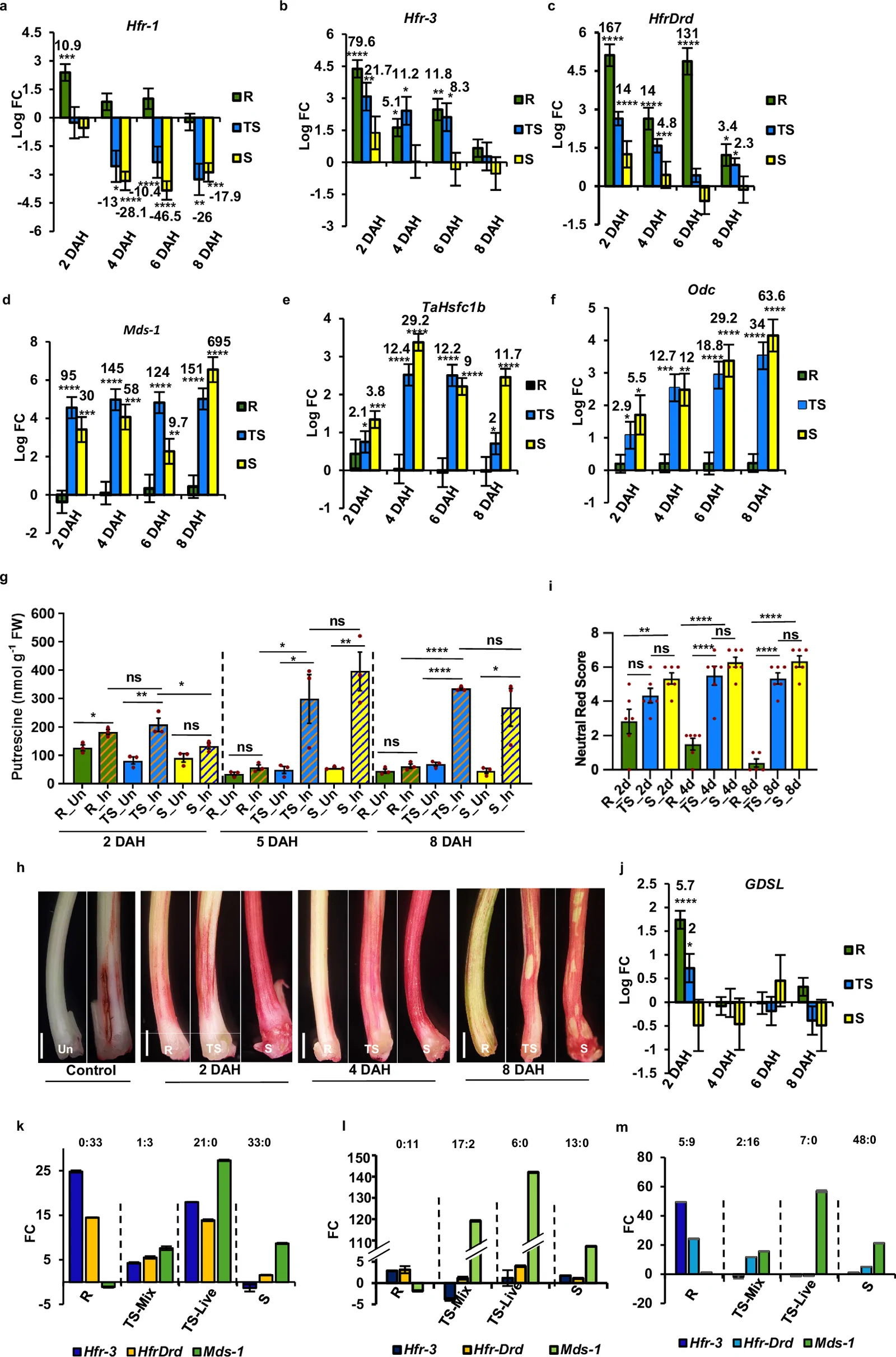
Molecular responses of durum wheat to Hessian fly larval feeding. Temporal expression of Hessian fly-defense and susceptibility associated genes in PI 519832 (resistant, R), Svevo (tolerant, TS), and PI 61188 (susceptible, S) wheat genotypes for **a***Hfr-1* (Hessian fly response gene 1). **b** *Hfr-3* (Hessian fly-responsive gene 3). **c** *HfrDrd* (Hessian fly-responsive disease resistant dirigent-like protein). **d** *Mds-1* (*Mayetiola destructor* gene 1). **e***TaHsfC1b* (Heat shock transcription factor). **f** *Odc* (Ornithine decarboxylase) at 2, 4, 6, and 8 DAH (days after egg hatch) time points. **g** Putrescine levels in uninfested (Un) and biotype L-infested (In) R, TS, and S plants at 2, 4, 6, and 8 DAH. **h** Changes in epidermal cell wall permeability as indicated by neutral red (NR) staining of biotype L-infested R, TS, and S plants at 2, 4, and 8 DAH. Representative uninfested TS plant was pin-pricked and used as positive control. Scale bars, 1 mm. **i** NR staining score^28^ in R, TS, and S plants at 2, 4, and 8 DAH (d). Statistical analysis (unpaired two-tailed t-test) was performed with GraphPad Prism (v10). Error bar represents standard error of mean. **j** Temporal expression of *GDSL* gene in R, TS, and S plants at 2, 4, 6, and 8 DAH. **k**–**m** Expression of *Hfr-3*, *HfrDrd,* and *Mds-1* in R, TS (TS-Mix and TS-Live), and S plants at 3 DAH. Data for (**a**–**f**, **j**) were collected from pooled crown (larval feeding site) tissue from *n* = 5 plants. Data represents three biological replicates and three averaged technical replicates per biological replicate and are provided in Supplementary Data 4. Data for (**k**–**m**) represent gene expression from 1 leaf sheath collected from three individual plants with three technical replicates per line and phenotype. Significant differences are indicated by asterisks as determined by ANOVA using the PROC MIXED procedure of SAS^63^). Individual data points are not shown because the means shown in the graphs are model-based lsmeans from the PROC MIXED analysis rather than simple averages. Only the significant fold changes (*p* ≤ 0.05, fold change ≥ 2 are indicated as numbers on top (upregulated genes) of the bars. *p ≤ 0.05, **p ≤ 0.01, ***p ≤ 0.001, and ****p ≤ 0.0001.

Because we observed larval antibiosis in TS plants, we evaluated the expression of select Hessian fly-responsive biomarker genes that are induced in resistant wheat plants. For this screening, our TS plant samples consisted of pooled leaf sheath 2 tissues (where larvae actively feed) from the TS-Mix and TS-Live phenotypes. As expected, the transcripts of *Hfr-1*^23^, a gene encoding a mannose-binding jacalin-like lectin causing larval antibiosis, were significantly upregulated in the *R* wheat at 2 DAH (10.9-fold; *p* = 0.001), but not in the TS plants (Fig. 3a). Interestingly, the *Hfr-1* transcripts were down-regulated several folds at 4, 6, and 8 DAH in the TS plants resembling the profile observed in the S plants (Fig. 3a). On the other hand, a significant increase in transcripts of *Hfr-3*^24^, a gene encoding a chitin-binding lectin with putative antibiosis property, was observed in the R and TS plants at 2, 4, and 6 DAH (Fig. 3b). *Hfr-3* transcript levels remained unchanged at all time points in the S plants (Fig. 3b). At 2 DAH (Fig. 3c), while *HfrDrd*, encoding for a dirigent protein that reinforces cell walls^25^, was upregulated in all three wheat genotypes (R, TS and S). The fold change of *HfrDrd* transcripts in R plants was much higher (167-fold; *p* ≤ 0.0001) compared to the TS and S plants that showed moderate (14-fold; *p* = 0.0003) and minimal (3.5-fold; *p* = 0.04) expression, respectively (Fig. 3c). *HfrDrd* transcript levels remained consistently high over time in both the R and TS plants, but not in S plants (Fig. 3c). These data demonstrate that the expression of Hessian fly-responsive resistance-associated defense genes in TS plants closely resemble expression in R plants.

### Hessian fly induces susceptibility factors in Svevo

The presence of live larvae in tolerant Svevo prompted us to evaluate how key biomarker genes associated with wheat susceptibility to Hessian fly expressed in these plants. *Mds-1* (*Mayetiola destructor susceptibility 1*) is a Hessian fly-responsive gene encoding for a small heat shock protein that serves as key biomarker for wheat susceptibility^26^. The transcripts of *Mds-1* increased significantly across all time points in TS and S plants, but not in R plants (Fig. 3d). Interestingly, unlike the susceptible plants, *Mds-1* transcript levels increased rapidly (between 2 and 6 DAH) in TS plants compared to the uninfested control (Fig. 3d). As expected, *Mds-1* levels in R seedlings did not vary between the infested and uninfested plants at any of the time points (Fig. 3d). In addition to *Mds-1*, increased transcript levels were observed for *TaHsfC1b*, a gene encoding a heat shock transcription factor, in the TS and S plants, while the R plants showed no differential expression (Fig. 3e). Increased transcripts of the susceptibility-associated *Odc* gene, encoding ornithine decarboxylase involved in the biosynthesis of polyamines^27^, were observed in both TS and S wheat lines (Fig. 3f). We also observed a corresponding increase in levels of the polyamine putrescine in the infested TS and S plants, but not in the infested R plants (Fig. 3g). Our data establishes that TS plants also resemble the S plants, with respect to expression of susceptibility-associated factors.

Since TS plants showed the presence of live larvae, which have the potential to break down cell wall polymers^5^, we assessed the temporal cell wall permeability and expression of genes encoding cell wall-associated proteins. Neutral red (NR) stained TS plants showed an intermediate phenotype, resembling the R plants at 2 DAH, and S plants at 4 and 8 DAH (Fig. 3h). At 2 DAH, TS plants stained as a smear, blush or solid lines, resembling the R phenotype, but covering more area of the crown tissue, giving it a higher, but non-significant, average NR rating as compared to either R or S plants (Fig. 3h, i). Furthermore, transcripts for the gene encoding a GDSL-motif lipase/hydrolase, an enzyme involved in altering cutin organization and increased cuticular permeability, showed a significant increase in R (5.7-fold) and TS (2-fold) plants at the early (2 DAH) time point (Fig. 3j), but decreased rapidly at later time points, mirroring the temporal profile of transient permeability in resistant wheat cells during larval attack^28^. No significant increase in abundance of this transcript was detected in the S plants at any of the time points (Fig. 3j). Temporal expression of 16 previously characterized^5^ wall-associated genes involved in cell wall fortification and strengthening was assessed (Supplementary Fig. 3). Three genes encoding for sucrose synthase (SuSy), glycosyltransferase (GT), and xylanase inhibitor (XI) showed significant up-regulation in both the TS and S infested plants (Supplementary Fig. 3), while the remaining 13 cell wall-associated genes showed significant down-regulation in the infested TS and S plants at all time points (Supplementary Fig. 3). None of the cell wall proteins were differentially expressed in the infested R plants at any of the time points (Supplementary Fig. 3).

To ascertain whether the resistance- and susceptibility-associated gene response was similar between TS-Live plants that had live larvae (LL) only, and TS-Mix plants that had a mix of LL and dead larvae (DL), we further evaluated transcript levels of *Hfr-3*, *HfrDrd*, and *Mds-1* in individual plants with the respective phenotypes. Figure 3k–m displays gene expression results for three individual leaf sheaths for each phenotype. Although we did observe a higher variability in transcript abundance in the individual sheaths (Fig. 3k–m) as compared to the pooled sheaths (Fig. 3b–d), there were similar expression profiles for the genes in all three sheaths per each phenotype collected from individual plants. Similar to the response in S plants, the individual sheaths from all three plants showed significantly higher *Mds-1* transcripts in both the TS-Mix and TS-Live plants (ranging from 8 to 43-fold) (Fig. 3k–m). Interestingly, the *Mds-1* transcript levels in all three TS-Live replicates were significantly higher than all TS-Mix and S plants (Fig. 3k–m). Transcript profiles for *HfrDrd* in the TS-Mix and TS-Live plants resembled the R plants more than the S plants (Fig. 3k–m). As expected, *Hfr-3* was upregulated consistently in the R plants, while only one sheath in each of the TS-Mix and TS-Live phenotypes showed increased *Hfr-3* transcripts (Fig. 3k–m), possibly reflecting the variability in infestation levels between different sheaths. Thus, our results clearly demonstrate an intermediate molecular response in TS plants with either live larvae alone, or mix of live and dead larvae, for the expression of both defense- and susceptibility-associated genes.

### Soft Svevo responses to Hessian fly strongly resemble Svevo

**Fig. 4:**
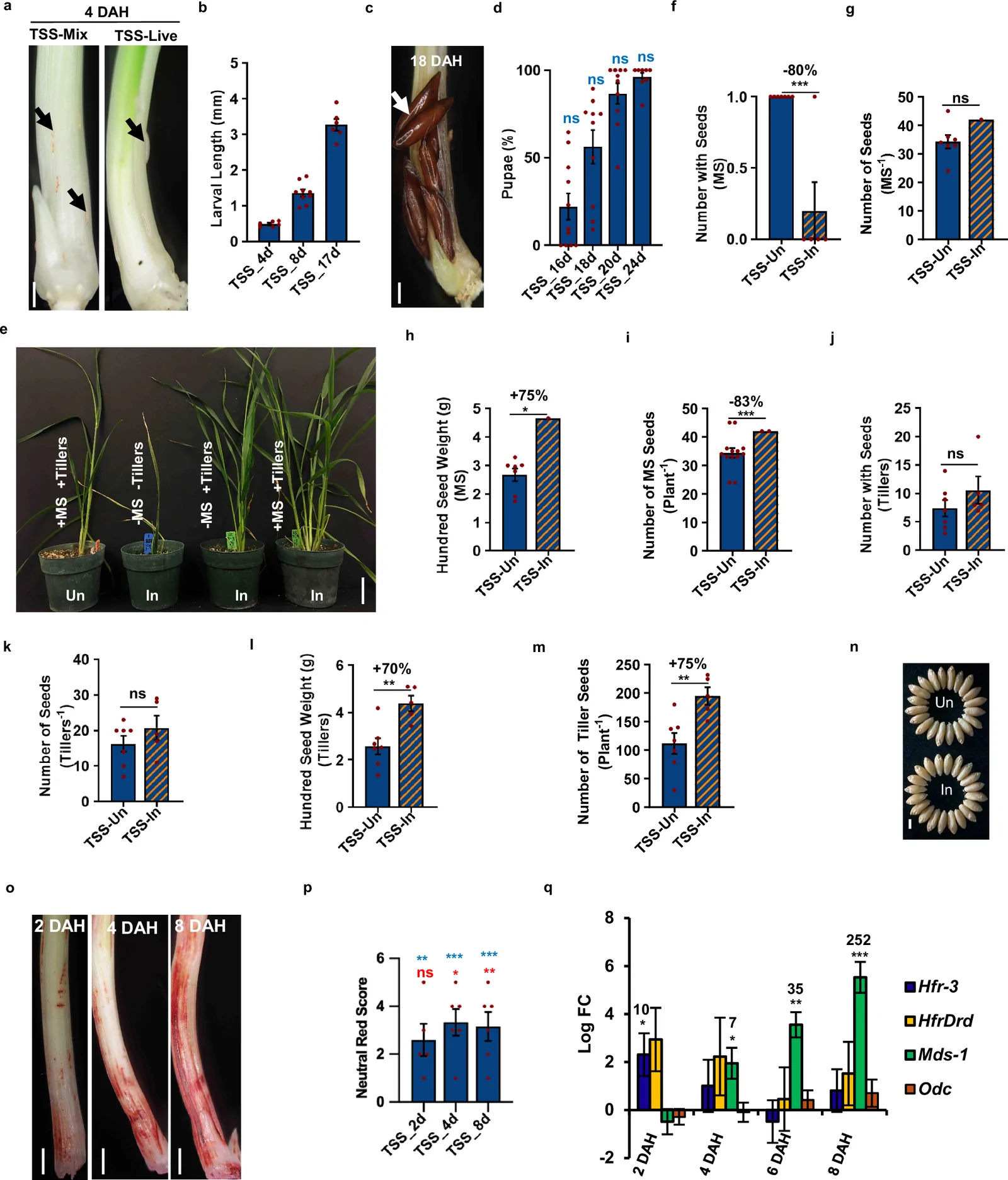
Phenotypic and molecular responses of Soft Svevo to Hessian fly larval feeding. **a** Representative photomicrograph of mix of dead (red, 1st instar) and live (white, 2nd instar) larvae on Soft Svevo (tolerant, TSS-Mix) and live (2nd instar) larvae on Soft Svevo (tolerant, TSS-Live) biotype L-infested plants at 4 DAH (days after egg hatch). Black arrows show larvae. Scale bars, 1 mm. **b** Larval length in TSS plants at 4, 8, and 17 DAH. **c** Representative photomicrograph of pupae (white arrows) on TSS plants at 18 DAH. **d** Percentage (%) pupae in TSS plants at 16, 18, 20, and 24 DAH (d). Statistical comparisons for the larval data were made using data from R (red asterisk) and S (blue asterisk) plants shown in Fig. 1. **e** Representative pot photos of Un and In TSS genotype showing variable phenotypes with (“+”) or without (“−”) MS (main stem; primary Hessian fly-infested stem) and tillers. Scale bar, 9 cm. Yield data were collected from Un and In TSS plants for **f** number of MS with seeds. **g** Number of seeds per MS. **h** Hundred seed weight (g) from MS. **i** Avg. number of seeds per plant. **j** Number of tillers with seeds. **k** Number of seeds per tiller. **l** Hundred seed weight (g) from tillers. **m** Number of tiller seeds per plant. **n** Representative photo of seeds harvested from uninfested (Un) and infested (In) TSS plants. Scale bar, 0.5 cm; **o** changes in epidermal cell wall permeability as indicated by neutral red (NR) staining of biotype L-infested TSS plants at 2, 4, and 8 DAH. Scale bars, 1 mm. **p** NR staining score^28^ in TSS plants at 2, 4, and 8 DAH (d); statistical analysis (unpaired two-tailed t-test) was performed with GraphPad Prism (v10). The number (*n*) of replicates for each experiment are provided in Supplementary Data 5. Each bar represents the standard error of mean. *p ≤ 0.05, **p ≤ 0.01, ***p ≤ 0.001, and ****p ≤ 0.0001. **q** Temporal expression of *Hfr-3* (Hessian fly responsive-3); *HfrDrd* (Hessian fly-responsive disease resistance dirigent-like protein); *Mds-1* (*Mayetiola destructor* gene 1); *Odc* (*Ornithine decarboxylase*) in biotype L-infested TSS seedlings at 2, 4, 6, and 8 DAH. Data are shown as logarithmic fold change (log_e_FC) of infested vs uninfested control at each time point, representing three biological replicates and three averaged technical replicates per biological replicate. Significant differences are indicated by asterisks as determined by ANOVA using the PROC MIXED procedure of SAS^63^. Individual data points are not shown because the means shown in the graphs are model-based lsmeans from the PROC MIXED analysis rather than simple averages. Only the significant fold changes (*p* ≤ 0.05, fold change ≥ 2) are indicated as numbers on top (upregulated genes) of the bars.

The elite cultivar Soft Svevo (designated as TSS) has soft kernels and is a preferred durum wheat line as it does not require specialized milling that limits end-product utilization^29^. We evaluated the phenotypic and molecular responses of Soft Svevo compared to the R and S plants assessed above. Similar to TS wheat, TSS plants showed a dual phenotype (~50% TSS-Mix and ~50% TSS-Live) in response to biotype L infestation (Fig. 4a and Table 2) and other biotypes (Table 2). We quantitatively compared (unpaired two-tailed t-test) the phenotypic traits for larval length, % pupae, and NR score in the TSS line (Fig. 4) with the data in the previous sections for R and S lines (Fig. 1). Larvae feeding on TSS plants were significantly longer (*p* ≤ 0.001) than the larvae on the R plants, but smaller than the S plants from 4 to 8 DAH (Fig. 4b). However, by 17 DAH, the TSS larval length was comparable to the larvae feeding on the S plants (Fig. 4b), with larvae pupating at around the same time as on S plants (Fig. 4c, d). At 22 DAH, 72% (5/7) of biotype L-infested TSS plants were stunted with significantly smaller lengths observed for ligules 3 (*p* ≤ 0.0001), 4 (*p* ≤ 0.0001), and 5 (*p* = 0.003), and leaves 4 (*p* = 0.008), 5 (*p* ≤ 0.0001), and 6 (*p* = 0.012), compared to the uninfested controls (Supplementary Fig. 1g, h).

**Table 2:** Phenotypic response of Soft Svevo to Hessian fly

Biotype	# plants	Avg. larvae	# plants (#LL)	# plants (#DL)	# plants mixed (#LL: #DL)	% plants (mixed response)	% DL
L	58	10	25 (269)	5 (33)	28 (223:65)	48.3	19
C	6	17	2 (38)	0	4 (57:4)	66.6	4
B	10	7	9 (56)	0	1(16:1)	10	1
O	10	7	3 (16)	1 (7)	6 (38:17)	60	24
D	10	7	7 (49)	0	2 (13:3)	20	5
*vH13*	9	13	8 (96)	0	1 (83:10)	11.1	2

# plants: total number of plants evaluated.

Avg. larvae: average number of larvae per plant.

# plants (#LL): number of plants harboring only live larvae (LL). The total number of LL from all plants are given in parentheses.

# plants (#DL): number of plants harboring only dead larvae (DL). The total number of DL from all plants are given in parentheses.

# plants mixed (#LL:#DL): number of plants harboring both LL and DL. The total numbers of LL and DL from all plants are given in parentheses.

% plants (mixed response): percentage of plants showing mixed response harboring both LL and DL.

% DL: percentage of DL in all plants.

Resembling the TS plants, biotype L-infested TSS plants showed variable phenotypes, including (i) 22% (2/9) of the plants lacking both the MS and tillers (−MS −Tillers); (ii) 56% (5/9) of the plants having tillers that produce seeds but lacking MS (−MS +Tillers); and (iii) 22% (2/9) plants having MS and tillers with both producing seeds (+MS +Tillers) (Fig. 4e). Resembling the TS plants, all infested TSS plants except the 22% that produced no seeds, including the ones with stunted ligules, leaves and −MS (Supplementary Fig. 1e, f), produced yield comparable (unpaired two-tailed t-test) to the uninfested plants with many having increased seed number and hundred seed weight (Fig. 4f–m).

Although there was a significant decrease in the number of MS with seeds (−80%; *p* = 0.007; Fig. 4f), the average number of seeds per MS remained comparable to uninfested plants (Fig. 4g), while the weight of seeds increased (+75%, *p* = 0.02*)* significantly (Fig. 4h). With respect to yield from tillers, the number with seeds and average number of seeds per tiller was comparable to the controls, while seed weight increased by 70% (*p* = 0.003) (Fig. 4j–m). The increase in total number of seeds is attributed significantly to the tillers (+75%, *p* = 0.008) compared to total number produced by MS (−83%; *p* < 0.01; Fig. 4i). Representative seed photos revealed no visual differences in the quality of seeds harvested from uninfested and infested plants (Fig. 4n). Cell wall permeability profiles in TSS plants (Fig. 4o) were similar to R wheat (Fig. 3h) at 2 DAH and by 8 DAH was still significantly less than S plants (Fig. 3h). Although the permeability profile was similar to the TS plants (Fig. 3h), the staining intensity was significantly lower in TSS plants at 4 and 8 DAH time points (Fig. 4p) compared to the TS plants (Fig. 3i). TSS plants showed increased transcripts of both the defense and susceptibility-associated Hessian fly biomarker genes (*Hfr-3* and *Mds-1*; Fig. 4q) resembling the expression profiles observed in the TS plants (Fig. 3b, d). *Hfr-3* levels were higher (10-fold; *p* = 0.03) at 2 DAH, while *Mds-1* was expressed higher by 6 DAH (35-fold; *p* = 0.0005) and 8 DAH (252-fold; *p* = 0.0001) (Fig. 4q). Unlike the TS plants, no significant change was observed for *HfrDrd* and *Odc*, typically associated with defense and susceptibility, respectively, in TSS plants over the time course (Fig. 4q). Our data demonstrate that the phenotypic and molecular responses of Soft Svevo (TSS) plants to Hessian fly infestation, resemble the tolerance responses observed in Svevo wheat.

### Hessian fly infestation induces metabolites in tolerant wheat

Primary and secondary metabolites play a critical role in the plant’s response to stress. We conducted metabolome profiling to identify metabolites potentially involved during wheat tolerance to Hessian fly. Untargeted metabolomic profiling of R, TS, TSS, and S genotypes identified 410 metabolites with confirmed MS/MS reference spectra, 4295 metabolites annotated based on accurate mass and retention time without MS/MS confirmation, and 3470 metabolites that remained unannotated. For this study, we used the 410 metabolites with reference spectra that were differentially accumulated in the wheat genotypes (Supplementary Data 1). A distinct demarcation between infested versus uninfested samples, and the TS/TSS wheat compared to the R and S wheat lines, was observed among the 410 metabolites identified (Supplementary Data 1 and Supplementary Fig. 4a). Of these, 162 were differentially accumulated metabolites (DAMs) (Supplementary Data 1) with the TS and TSS wheat lines showing intermediate number of accumulated metabolites between R and S wheat lines but a higher number of metabolites with decreased levels (Supplementary Fig. 4b). Unlike the R wheat line, the TS, TSS and S wheat lines showed increased levels of the DAMs from 4 to 8 days post-infestation (Supplementary Fig. 4c, d). Additionally, TS and TSS shared a higher number of DAMs with S wheat (Supplementary Fig. 4e, f).

**Fig. 5:**
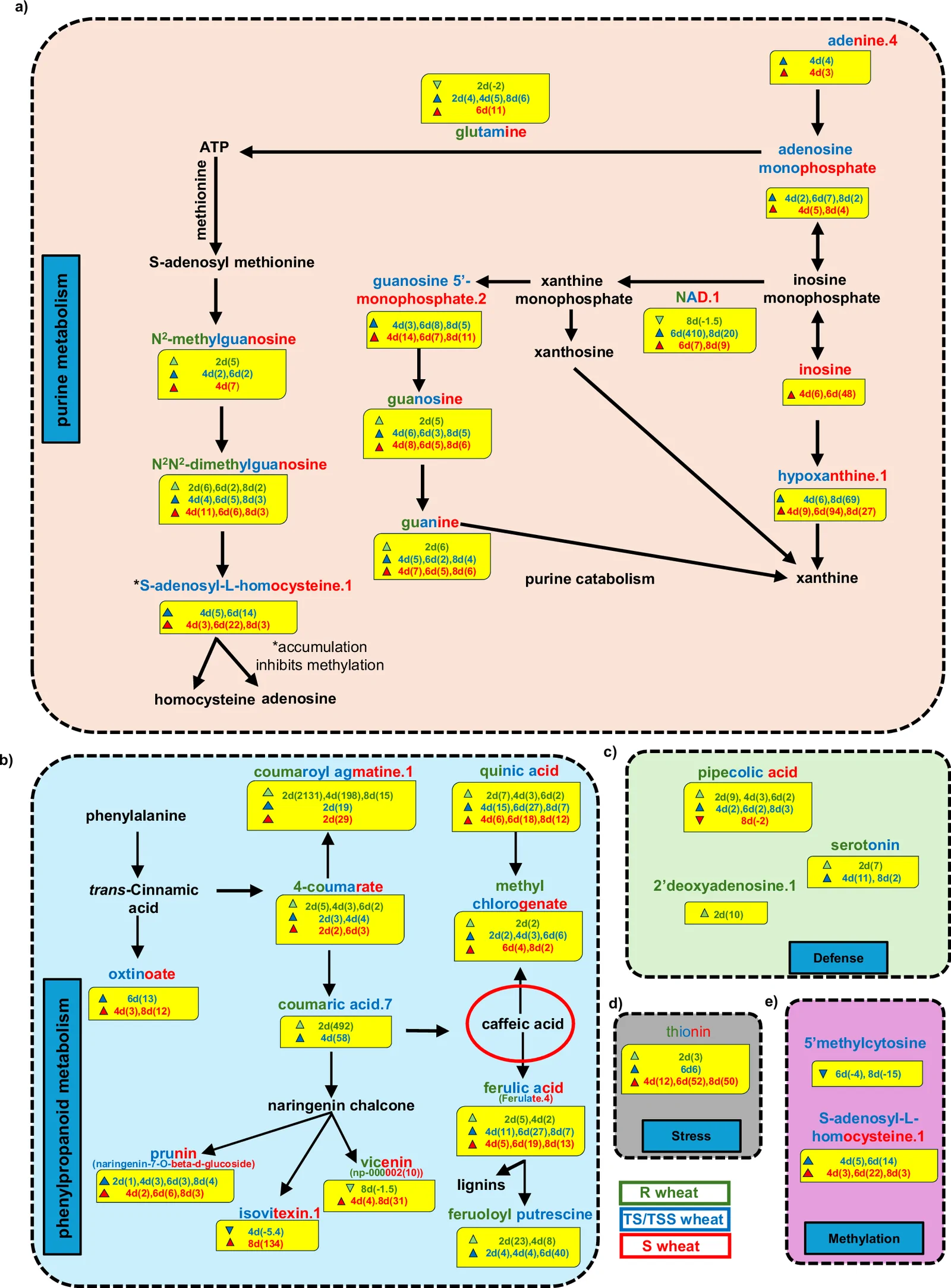
Functional classification of differentially accumulated metabolites in durum wheat to Hessian fly larval feeding. Overview of select DAMs identified from biotype L-infested PI 519832 (R, resistant); Svevo (TS, tolerant); Soft Svevo (TSS,) and PI 61188 (S, susceptible) wheat genotypes at 2, 4, 6, and 8 DAH (days after egg hatch) time points that were grouped based on functional annotation: **a** purine metabolism. **b** Phenylpropanoid metabolism. **c** Defense. **d** Stress. **e** Methylation. Metabolite names are highlighted in different colors (green: R; blue: TS; red: S) to indicate the genotype/s showing differential accumulation. Metabolites represented in black indicate no significant change in levels between infested vs uninfested wheat samples. Significant fold changes for each genotype (given within parentheses) and time point (d indicates days after egg hatch) are provided in the yellow boxes. ▲ indicates increased and ▼ indicates decreased metabolites. For the two tolerant genotypes, TS and TSS, the genotype showing the highest fold change for a particular time point was chosen for representation.

For comprehensive visualization, the DAMs are represented as heatmaps with significant fold changes listed for the infested sample compared to the uninfested control (Supplementary Figs. 5–8). The profiles for stereoisomers for some metabolites (represented with sequential numbers) differed (Supplementary Figs. 5–8), signifying high specificity in metabolite induction. Resembling the S wheat line, the TS/TSS lines were significantly enriched for purine metabolism with most metabolites showing increased levels at 4-8 DAH (Fig. 5a). These included several precursors and intermediates of the purine building block, and de novo/salvage pathway components including N^2^ methylguanosine, adenosine monophosphate, guanosine monophosphate, guanine, and hypoxanthine (Fig. 5a). Three metabolites (guanosine, guanine, and N^2^ methylguanosine) showed rapid (2 DAH only) elevation in the R plants (Fig. 5a). Metabolites from the phenylpropanoid metabolism were also significantly enriched in TS/TSS wheat (Fig. 5b). Unlike purine metabolism, many of the metabolites associated with the phenylpropanoid pathway were also accumulated in the R plants (Fig. 5b). The metabolite accumulation levels in the TS/TSS plants either resembled the resistant or the susceptible wheat line. Notably, while several key metabolites upstream and downstream of caffeic acid (CA) showed increased levels in all three phenotypes, CA itself did not differentially accumulate in any of the infested wheat genotypes (Fig. 5b). The TS/TSS and R wheat lines also showed increased accumulation of pipecolic acid and serotonins, known to be involved in plant defense to insect herbivory (Fig. 5c). The metabolite 2'-deoxyadenosine.1, was only associated with the R wheat line at 2 DAH (10-fold, *p* ≤ 0.05) but not with the TS/TSS or S wheat lines (Fig. 5c). Increased level of thionin was observed in all four genotypes, however, these were up at different time points (Fig. 5d). Interestingly, the metabolites 5-methylcytosine and S-adenosyl-L-homocysteine.1, involved in DNA methylation, were differentially expressed in TS and TSS plants (Fig. 5e).

### Caffeic acid primes tolerance in wheat to Hessian fly

**Fig. 6:**
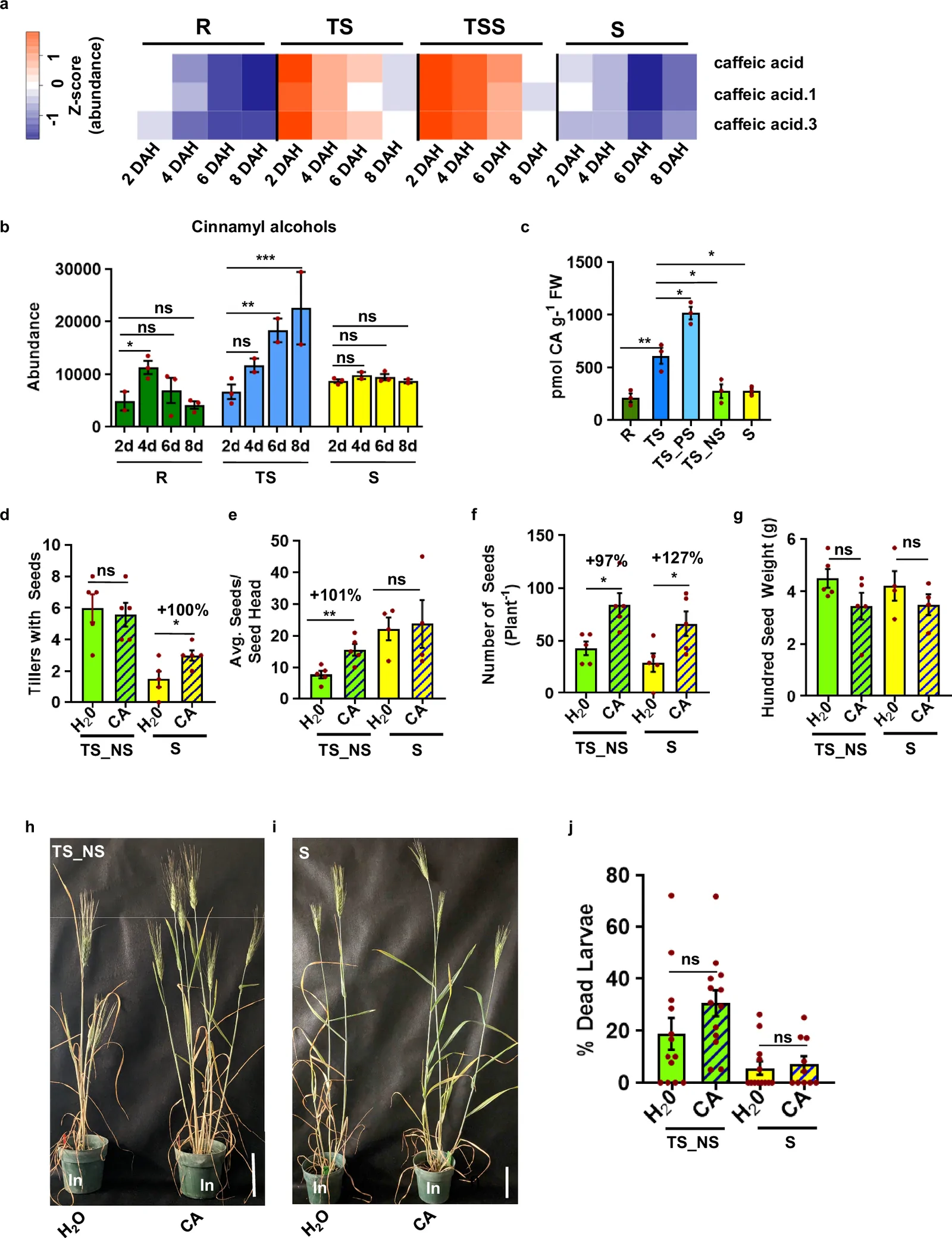
Effect of exogenous caffeic acid treatment on durum wheat to Hessian fly larval feeding. **a** Heatmap showing constitutive levels of three stereoisomers of caffeic acid (CA) in uninfested PI 519832 (R, resistant); Svevo (TS, tolerant); Soft Svevo (TSS), and PI 61188 (S, susceptible) wheat genotypes at 2, 4, 6, and 8 d (corresponding to days after egg hatch in the infested plants). Red indicates higher CA levels and blue indicates lower CA levels in each genotype and time point. Data represents Z-score abundance from three biological replicates. **b** Abundance of cinnamyl alcohols in biotype L-infested R, TS, and S plants at 2, 4, 6, and 8 DAH (days after egg-hatch). Lines above indicate the statistical significance in abundance levels at 4, 6, and 8 DAH time points compared with 2 DAH time point for a particular genotype. **c** Detection of CA in uninfested samples of R, TS, TS_PS (TS plants positively selected for MS survival trait from Fig. 2), TS_NS (TS plants negatively selected for MS survival trait from Fig. 2), and S wheat genotypes. **d–g** Yield data collected from biotype L-infested TS_NS and S plants treated with 2 mM CA (CA) or water (H_2_O) for **d** number of seed heads producing seed. **e** Avg. (average) seeds per seed head. **f** Number of seeds per plant. **g** Hundred seed weight (g) per plant. **h**,** i** Representative pot photos showing biotype L-infested CA- or H_2_O- treated plants. Scale bars, 4.5 cm. **j** Percentage (%) of dead (1st instar) larvae in H_2_O- and CA-treated TS_NS and S plants. Statistical analysis (unpaired two-tailed t-test) was performed using GraphPad Prism (v10). The number (*n*) of replicates for each experiment are provided in Supplementary Data 6. Error bars represent standard error of mean. *p ≤ 0.05, **p ≤ 0.01, ***p ≤ 0.001, and ****p ≤ 0.0001.

While caffeic acid (CA) was not differentially accumulated, we observed higher basal levels of CA in the uninfested TS and TSS wheat samples compared to either the R or S wheat lines (Fig. 6a). Further, we observed a significant increase in abundance of cinnamyl alcohols in TS plants by 6 (*p* = 0.007) and 8 (*p* < 0.0001) DAH from 2 DAH (Fig. 6b). In R plants, while there was a small but significant increase by 4 DAH (*p* = 0.039), by 6 and 8 DAH the abundance levels returned to the 2 DAH levels (Fig. 6b) corresponding with death of 1st-instar Hessian fly larvae. In contrast, the S plants showed no significant difference in cinnamyl alcohol levels between the time points (Fig. 6b). Caffeic acid is a key intermediate in the phenylpropanoid pathway, leading to the production of cinnamyl alcohols (monolignols) that undergo oxidative polymerization to form lignins in plants, and help with fortifying the cell wall during biotic stress. The higher constitutive CA levels and increase in the precursors of lignin led us to hypothesize that they may be directly involved in tolerance by priming the plants to tolerate insect herbivory. To test our hypothesis, we performed targeted metabolomic profiling to determine the constitutive levels of CA. Uninfested TS plants had around 2- to 3-fold higher basal CA levels compared to R, S and TS_NS (TS plants negatively selected for MS survival trait) plants (Fig. 6c). Interestingly, the TS_PS plants (TS plants positively selected for MS trait) showed 1.6-fold (*p* = 0.013) higher basal CA levels compared to the TS plants (Fig. 6c). To further substantiate our priming hypothesis, we exogenously treated TS_NS and S plants with CA and observed significant improvements in plant yield post-infestation (Fig. 6d–g). While the number of tillers and MS with seeds were comparable between infested and uninfested CA-treated TS_NS plants there was a significant increase in average seed number per seed head (+101%, *p* = 0.007) and seed number (+97%; *p* = 0.0113) per plant (Fig. 6d, e). The CA-treated S plants also exhibited increase in number of seeds per plant (+127%; *p* ≤ 0.01) compared to the water-treated controls (Fig. 6f). Plant growth between CA-treated and water-treated controls post-infestation was comparable (Fig. 6h, i). There was no significant difference in the percentage of dead 1st-instar larvae between the CA-treated and water-treated TS_NS or S infested plants (Fig. 6j). These data confirm our hypothesis that CA primes tolerance in wheat.

## Discussion

Durum wheat is widely used for pasta production^30^. Among elite cultivars, Svevo is known for its high yield, excellent pasta-making qualities, and strong resistance to biotic and abiotic stressors, including fungal pathogens, drought, heat, and salinity^31–34^. Additionally, durum wheat is better adapted to the hot, arid growing regions of the United States than the more frequently grown bread wheat cultivars^29^. Soft Svevo was developed as a new cultivar of durum wheat with a soft texture and better milling properties resembling bread wheat, further expanding its commercial market beyond traditional pasta. Soft Svevo, like Svevo wheat, also exhibits resistance to various biotic and abiotic stresses^29^. In this study, we demonstrate that both Svevo and Soft Svevo are tolerant to Hessian fly.

Our study on the careful phenotypic characterization of tolerant elite durum wheat lines addresses the critical knowledge gap associated with the understanding of plant tolerance to insect pests. We identified two major phenotypes in tolerant wheat that bridged the resistant and susceptible plant phenotypes. We clearly demonstrated that these phenotypes are not associated with segregating resistance, as dissections of more than one hundred infested plants showed that the tolerance is always associated with a dual phenotypic response of all larvae surviving (TS-Live) or a mix of both dead and live larvae (TS-Mix) on the same plant, but never with the response of all dead larvae, typical in resistant wheat lines. Unlike the resistant plants, the dissection of infested tolerant plants revealed fully pupated larvae, with around 14-20% dead larvae seen at early timepoints. However, this minimal penalty in larval development does not impede the successful completion of the life cycle, with viable adult Hessian flies emerging that can infest susceptible wheat. Unlike susceptible wheat, despite the presence of 76-95% live larvae completing their life cycles, tolerant Svevo and Soft Svevo plants display yields comparable and often higher than the uninfested plants despite around 20% of infested tolerant plants producing no seeds, and about half of the infested plants exhibiting stunted main stem growth. Most of the tolerant plants that had stunted leaves 4-6 were still able to produce tillers with seed numbers and weight at levels comparable, and often better than the uninfested controls, sustaining no yield penalties due to Hessian fly infestation. Our findings clearly demonstrate that, unlike resistance and susceptibility, tolerance in Svevo and Soft Svevo not only maintains larval fitness but also has increased yields under both infested and uninfested conditions. It is possible that tolerant plants compensate for herbivory to maintain growth and fitness or overcompensate and perform better^35^. Compensatory growth is a form of tolerance that allows the plants to mitigate damage, sometimes exceeding pre damage biomass levels. Interestingly, selection for and against the primary Hessian fly-infested main stem (MS) survival significantly increased and decreased, respectively, the total seed numbers in uninfested TS_PS and TS_NS plants, compared to the uninfested, unselected TS plants. This suggests that the MS survival after infestation trait could be linked to genetic factors affecting yield even in the absence of Hessian fly infestation.

The Hessian fly induces wheat plant susceptibility by injecting salivary effectors that play important roles in host plant manipulation^5,36^. The resistant wheat via the *R* gene-mediated resistance machinery, reinforces cell wall defense and triggers defense pathways and molecules, including insecticidal lectins, reactive oxygen species, and secondary metabolites to counter the insect effectors^23,25,37–40^. In contrast, in the absence of *R* gene-mediated resistance, the larval effectors reprogram the susceptible host plant physiology, suppress defense genes, induce the formation of a nutritive tissue rich in amino acids, sugars, and polyamines, and increase cell wall permeability that benefit the developing larvae^27,28,41–43^.

We discovered that, surprisingly, tolerant wheat exhibits an intermediate molecular response that resembles both resistant and susceptible wheat. Infested tolerant wheat plants (TS-Mix and TS-Live) showed significantly elevated expression of Hessian fly-responsive resistance (*Hfr-3* and *HfrDrd*) and susceptibility-associated (*Mds-1* and *Odc*) genes, which serve as biomarkers for resistant and susceptible wheat, respectively. Notably, tolerant plants did not induce *Hfr-1*, another biomarker gene linked to wheat resistance, encoding a mannose-specific jacalin lectin with insecticidal and antifeedant properties leading to larval antibiosis^23,37^. Likely, the resistance-linked gene *Hfr-3*, encoding a chitin-binding lectin with proposed anti-nutritional activity leading to larval starvation^24^, is a contributing factor in the death of larvae on the TS-Mix plants. Damage caused by the live larvae may be minimized by expression of the *HfrDrd* gene that encodes a dirigent protein involved in regulating lignin and lignan biosynthesis, contributing to structural fortification of the wheat cell wall in response to Hessian fly infestation^25^. These results suggest that *Hfr-1* expression is either specifically linked to the *R*-gene resistance response, or its suppression may be an adaptive feature in the tolerant plants to prevent larval antibiosis associated with a typical resistance response. In contrast, the expression of *Hfr-3* and *HfrDrd* is required for general plant defense that can make the environment less favorable for the larvae. The tolerant wheat resembled susceptible wheat in the increased expression of both *Mds-1* and its putative transcriptional regulator, *TaHsfC1b*. Further, increase in putrescine levels, intense cell wall permeability, and suppression of cell wall genes in tolerant wheat clearly indicate a stronger resemblance to susceptible wheat, creating a molecular environment conducive for the larvae to procure the necessary nutrients for development. Our results suggest that although there is a defense response initiated as an early defense strategy that is detrimental to some larvae, as evident by 5–20% larval death in infested TS-Mix plants, the induction of susceptibility factors likely allow the surviving larvae to complete their development, imposing little to no selection pressure on the insect population. These results support an intermediate physical and molecular response in TS plants, thus resembling both resistant and susceptible wheat. Our findings further revealed that increased susceptibility factors did not have any effect on the tolerant plant yields, unlike either the resistant or susceptible plants that show insect and yield penalties, respectively. The phenotypic and molecular responses clearly indicate that mechanisms in tolerant wheat are distinctive from either resistant or susceptible wheat. Screening wheat accessions for the dual phenotypes we observed on tolerant wheat, patterns of *Hfr-3* and *Mds-1* expression, and thorough yield studies comparing uninfested and infested plants of an individual wheat line may serve as reliable phenotypic and molecular biomarkers for identifying wheat Hessian fly tolerance.

Plant primary and secondary metabolites are key players in plant-insect interactions^44^. Primary metabolites, including proteins, amino acids, carbohydrates, and lipids, play an essential role in plant growth and development, while secondary metabolites are important for plant defense against insect herbivory^45^. Metabolomic profiling revealed significant accumulation of purine-based metabolites temporally in the tolerant wheat, resembling the profile observed in the susceptible wheat. In plants, purine nucleotides participate in many biochemical processes that are involved in the synthesis of primary products, including sucrose, polysaccharides, and phospholipids, and therefore, are of fundamental importance in plant growth and development^46^. Insects are unable to directly digest plant purines but instead use bacterial endosymbionts to break down purines that allow them to utilize nitrogen, which is considered the most limiting nutrient element for herbivorous insects^47^. The microbiota found in the Hessian fly gut are essential for larval survival on wheat seedlings^48^. Our results suggest that enrichment of purine metabolism in tolerant wheat directly benefits plant growth, and indirectly benefits insect development.

Secondary metabolite profiles from the phenylpropanoid metabolism shared resemblances with both the resistant and susceptible plants, suggesting that they may possibly be contributing to both specific plant defense and general stress responses to larval feeding^44^. Higher basal levels of CA in the tolerant plants, compared to R and S plants, as well as increased plant yield after exogenous CA application in the infested susceptible and tolerant plants, negatively selected for MS survival, suggest that CA contributes to wheat Hessian fly tolerance. Additionally, the tolerant wheat plants showed a steady increase in cinnamyl alcohols, a family of monolignols. In contrast, in the resistant wheat, the levels increased till 4 DAH and then decreased, coinciding with larval death by 5 DAH. The susceptible wheat showed no difference in levels of cinnamyl alcohols between the time points.

Caffeic acid is the key metabolite in the phenylpropanoid pathway in plants that generates a variety of aromatic compounds, including cinnamyl alcohols, which are building blocks of lignin, a polymer that strengthens plant cell walls. It is well documented that priming of plants with natural compounds leads to a physiological state that enables plants to respond rapidly and robustly to environmental stress^49,50^. It is proposed that priming keeps plants at a heightened state of readiness, which involves molecular changes, such as accumulation of signaling molecules; epigenetic modifications that allow plants to memorize stress encounters; and rapid deployment of defense proteins that enables rapid and efficient response to a stress. These priming metabolites are proposed to play a crucial role in activating plants’ own defense mechanism to combat unforeseen stressors and enhancing crop resilience^51^. Increased CA positively correlates with improved tolerance to oxidative and other abiotic stress^52^. We propose that priming wheat plants with CA alleviates Hessian fly stress and promotes seedling growth by fortifying the plant cell wall and triggering activation of other defense or stress metabolites downstream, contributing to plant tolerance. The higher transcripts of *HfrDrd*, a dirigent protein involved in lignin synthesis, in the tolerant wheat further supports our hypothesis that the plant defense measures are augmented in tolerant plants.

We also observed increased levels of pipecolic acid and serotonins in the infested resistant and tolerant wheat, suggesting their role in plant defense to Hessian fly larval attack. Pipecolic acid is involved in systemic acquired resistance (SAR) during plant-pathogen interactions^53,54^ and abiotic stress adaptation^55^, and serotonins are vital for plant growth and stress response^56^. Pipecolic acid is known to act as a powerful immune priming agent by enhancing transcriptional reactivity of defense genes and creating a long-lasting immune memory during stress-free periods. While there is no evidence of SAR during wheat-Hessian fly interactions, it is possible that activation of pipecolic acid following Hessian fly infestation prepares the plant to defend when attacked later. The activation of serotonins in tolerant plants suggest early defense against insect stress and may act as growth regulators. Our data supports the role of metabolites including caffeic acid, pipecolic acid and serotonins in plant tolerance to mitigate feeding pressure from the developing Hessian fly larvae. However, additional studies are needed to pinpoint the specific role of these metabolites during wheat tolerance and resistance to Hessian fly.

Metabolite profiling also revealed a significant decrease in levels of 5-methylcytosine, involved in DNA methylation, exclusively in tolerant wheat. Additionally, we found increased accumulation S-adenosyl-L-homocysteine.1 in tolerant wheat, which is known to inhibit methylation^57^. Our findings suggest hypomethylation as a possible mechanism for regulating robust and rapid expression of stress-responsive genes, promoting plant immunity to tolerate insect herbivory. Epigenetic priming is shown to be an adaptive plant strategy to enhance activation of induced defense mechanisms to fortify the plants through modulation of gene expression without altering the underlying DNA sequence under various stresses^58^. Detailed studies are required to validate the possible involvement of epigenetic mechanisms in regulating wheat tolerance to the Hessian fly.

Tolerant wheat essentially behaves similarly to susceptible wheat but carries unique adaptive molecular signatures that prime or regulate the plants, allowing them to tolerate insect herbivory and injury, distinguishing this response from the complete antibiosis seen in *R* gene-mediated resistance. The key to larval survival on tolerant plants is their ability to take up nutrients while simultaneously coping with defense molecules and metabolites. Taken together, there is minimal penalty on insect fitness and, surprisingly, increased yield in the tolerant wheat plants. Tolerance in wheat to Hessian fly involves compensatory growth that contributes to plant yield as a coping mechanism to mitigate insect damage. Additionally, evidence in this study suggests that some traits linked to Hessian fly tolerance can increase yield even in the absence of Hessian fly infestation. Our experiments were conducted under controlled environmental conditions, hence it is important to note here that the effect of genotypes, environment, and management is expected to affect the performance and yield of Hessian fly-infested tolerant plants. Additionally, extensive field investigations are warranted to validate the effectiveness of tolerance as a mitigation strategy for the management and control of Hessian fly. Identifying tolerance in elite lines with inherent beneficial traits is a lucrative strategy for wheat growers and producers without the years of effort necessary to identify and breed the required trait into elite wheat lines^59^. This strategy, if implemented, can potentially circumvent the rapid progression of more virulent insect-field populations, delay the breakdown of native resistance in the field, and reduce the dependence on innate insect-resistance alone in wheat, thereby contributing to sustainable wheat production for growing global population. However, further dissection of each molecular component underlying tolerance is required for efficient manipulation of these adaptive signatures. Tolerance, along with innate resistance, when employed in integrated pest management systems, can potentially reduce significant yield losses, ensuring wheat crop sustainability.

## Methods

### Insect material

The Hessian fly (*Mayetiola destructor*) stocks for biotypes B, C, D, L, O, and *vH13* used in the current study were maintained in diapause in 4 °C at the USDA-ARS Crop Production & Pest Control Research Unit, West Lafayette, IN, as described^60^. For each experiment, the purity of Hessian fly stocks was tested by scoring the response of wheat cultivars “Monon,” “Magnum,” “Caldwell,” and “Seneca,” harboring Hessian fly resistance genes *H3*, *H5*, *H6,* and *H7H8*, respectively, to the insect infestation. The purity of *vH13* Hessian fly stock was assessed by infesting wheat lines “Iris” and “Molly” harboring the *H9* and *H13* resistance genes, respectively.

### Wheat infestation and phenotyping

The durum wheat *Triticum turgidum* L. subsp. *durum* (Desf.) van Slageren accessions used in this study include: PI 519832, PI 61188, Svevo, and Soft Svevo. Seeds of PI 519832 and PI 61188 were obtained from USDA-ARS National Small Grains Collection (Aberdeen, ID), Svevo seeds were obtained from Western Regional Center (Albany, CA), and Soft Svevo seeds were procured from USDA-ARS Western Quality Lab (Pullman, WA). The wheat lines were grown in 5 pots per genotype (15 plants per pot) and evaluated for their phenotypic response to Hessian fly biotypes (B, C, D, L, O, and *vH13*) at 18 °C as described^61^. The treatments included Hessian fly-infested and uninfested plants. Individual plants were considered as replicates for each experiment, and data analyzed at the plant level.

Between 4 and 8 days after egg hatch (DAH), the infested wheat seedlings were dissected, and the number of dead, red larvae (DL; 1st instars) and white, live larvae (LL; 1st or 2nd instars) were recorded. The larval and pupal development was assessed by capturing images on SZX2 stereomicroscope with a DP27 camera system (Olympus America Inc., Center Valley, PA). The larval lengths were measured using the Olympus Cell Sens Standard Software. Whole pot photos were also taken with a Nikon Nikkor AF-S (Nikon Americas Inc., Melville, NY) camera. The ligule and leaf length measurements from individual plants were taken on the primary Hessian fly-infested main stem (MS) as described^62^.

### Yield data

The yield data were collected from the biotype L-infested and uninfested wheat plants. The plants were allowed to grow until they produced seed and matured. Data were collected from both the main stem and tillers for each plant. To distinguish the main stem from tillers following infestation, the primary Hessian fly-infested stems were marked with a black permanent marker. The seed heads from the main stems and tillers were collected and allowed to dry overnight in an incubator set at 37 °C. Each seed head was threshed, and the number of seeds per seed head was counted and weighed. For each plant, the 100-seed weight was calculated for MS or tillers by dividing the total seeds weight (g) by the number of seeds, multiplied by 100. The yield data for tolerant wheat lines (Svevo and Soft Svevo) was computed from all the phenotypes (TS-Mix and TS-Live). The statistical analysis was performed using unpaired two-tailed t-test using GraphPad Prism (v10). Pearson’s correlation (*r*) analysis and corresponding two-tailed *p*-values were calculated using Microsoft Excel software to assess the significance of yield parameter (number of seeds) between plants having MS (primary Hessian fly infested main stem) but lacking tillers (MS -tillers) and plants harboring both MS and Tillers (MS +tillers).

### Main stem selection

Since the biotype L-infested Svevo wheat plants with main stem survival showed an increase in the total number of seeds and seed weight, we proceeded to select this trait in this wheat line. The selection was done for the presence and survival of main stem or absence of the main stem for four generations using traditional trait selection methods as detailed in the schematic representation prepared using BioRender Scientific Image and Illustration Software (Supplementary Fig. 2). The number of plants used in this study are included in Supplementary Fig. 2.

### Neutral red staining

The cell wall permeability in all the biotype L-infested durum wheat lines was determined by staining the plants with 0.1% (w/v) Neutral Red Stain (NR; Sigma-Aldrich, St. Louis, MO) at 2, 4, and 8 DAH as per^28^. Uninfested and pin-pricked wheat plants for each line (5–6 plants per wheat line per treatment) were used as negative and positive controls, respectively. Photomicrographs were taken with a DP27 camera on a SZX2 stereomicroscope (Olympus).

### Transcript profiling

Expression of select Hessian fly-responsive defense- and susceptibility-associated genes was evaluated and compared between the durum wheat accessions. The crown tissue from uninfested and biotype L-infested wheat seedlings was collected over a time course (2, 4, and 8 DAH), and gene expression carried out using quantitative real-time reverse transcription PCR (qRT-PCR) as described^61^. Sequences of primers used are given in Supplementary Data 2. The *T. aestivum* ribosomal 18S gene (NCBI Genbank Accession # AY049040) was used as endogenous reference gene for normalization of qRT-PCR data. Two sets of qRT-PCR experiments were performed: (i) using pooled crown tissue (5 plants per sample per replication) and (ii) single crown tissue (1 plant per sample). Data were collected from three biological replicates with three technical replications). Statistical analysis for transcript abundance was carried out, and logarithm-transformed values were determined by analysis of variance (ANOVA) using the PROC Mixed procedure of SAS Software version 9.4 as described^63^.

### Polyamine analysis

The temporal changes in levels of polyamines were determined in crown tissue collected^27^ from biotype L-infested and uninfested R, TS, and S wheat plants (10–12 plants per replicate) on 2, 5, and 8 DAH as described^64^. Data were collected from three biological replicates for each sample. Polyamine levels are expressed as nmols g^−1^ FW.

### Untargeted metabolite analysis

Untargeted metabolite analysis was performed using the uninfested and biotype L-infested wheat (R, TS, TSS, and S) crown tissue collected from 10 plants per biological replicate at 2, 4, 6, and 8 DAH time points. Three biological replicates were collected and analyzed at the Purdue University Metabolite Profiling Facility. Protein removal and sample extraction was performed by bead homogenization and solvent extraction. In summary, 100 mg of tissue was added to a Precellys CK28 tube and homogenized. Then 1000 μL of 80% methanol and 20% water was added, and homogenization repeated. Liquid was transferred to a microcentrifuge tube, followed by centrifugation at 10,000 × *g* for 8 min. The supernatant was transferred to a microcentrifuge vial and evaporated to dryness in a vacuum concentrator. The remaining pellet was reconstituted in 75 μL of diluent composed of 95% water and 5% acetonitrile, containing 0.1% formic acid. The reconstituted sample was sonicated for 5 min, centrifuged at 16,000 × *g* for 8 min, and the supernatant was transferred to a HPLC autosampler vial. Separations were performed on an Agilent 1290 system (Agilent Technologies Inc., Palo Alto, CA), with a mobile phase flow rate of 0.30 mL/min. The metabolites were assayed with a 5 μL injection using a Waters Atlantis T3 column (3 µm, 2.1 × 150 mm) (Milford, MA), where mobile phase A and B were 0.1% formic acid in ddH_2_O and acetonitrile, respectively. Initial conditions were 100:0 A:B, held for 1.5 min, followed by a linear gradient to 80:20 at 24 min, then 5:95 at 31.5 min with an isocratic hold until 33.8 min. Column re-equilibration was performed by returning to 100:0 A:B at 35.3 min and holding until 42.8 min. The mass analysis was obtained in positive ionization mode using an Agilent 6546 Q-TOF mass spectrometer with ESI capillary voltage of +3.5 kV. MS/MS was performed in data dependent analysis (DDA) mode, with a range of 70–1000 m/z and fixed collision energies of 10, 20, and 40 eV. Mass accuracy was improved by infusing Agilent Reference Mass Correction Solution (G1969-85001). Peak deconvolution, integration, and statistical analysis were performed using MS-DIAL (v. 4.9) [https://systemsomicslab.github.io/compms/msdial/main.html]. Peak annotations were performed using the MassBank of North America MS/MS library, based on authentic standards (v. 17) [https://systemsomicslab.github.io/compms/msdial/main.html#MSP]. Mass tolerances for identification were 0.005 Da for MS1 and 0.01 Da for MS2. Data collection methods are provided in Supplementary Methods. The raw data were processed, and only the 410 known metabolites identified were selected for further analysis. The raw abundance data for the metabolites were log-transformed and normalized using Pareto-scaling^65^ for principal component analysis (PCA) to explore the statistical correlation between the genotypes and time points. To identify differentially accumulated metabolites (DAMs), a univariate statistical analysis of each infested versus control sample for a specific time point was performed. The metabolites were considered statistically significant DAMs for values having *p* ≤ 0.05 and log_2_ fold change ≥ 1^66–68^.

### Targeted metabolite analysis

As untargeted metabolomics profiling revealed enhanced levels of certain metabolites in Svevo plants, targeted metabolomics was performed with uninfested crown tissue (3 biological replicates with 10 plants per replicate) collected from R, S, TS, TS_PS, and TS_NS wheat accessions at the 2 days time point (corresponding to 2 DAH in infested wheat seedlings) and processed at Texas A&M University using the 4500 LC-MS/MS system (Sciex, Farmingham, MA) as described^69^.

### Caffeic acid treatment

The effect of caffeic acid (CA) on wheat yield was evaluated by treating Svevo plants negatively selected for MS survival trait (TS_NS) and susceptible PI 61188 wheat plants. Seeds were planted in 4-inch pots (5 seeds per pot) as described above. 75 mL of 2 mM CA (Indofine, Hillsborough, NJ) solution was applied directly to the soil twice: during seed planting and after germination. When the plants were at the 1-leaf stage, a day prior to infestation with biotype L Hessian fly, each pot (with 5 plants) was sprayed 5 times with 2 mM CA solution, and plants were allowed to dry. Tween 20 were added to all spray treatments at a final concentration of 0.025% as a surfactant. The plants were infested as described above and checked for infestation levels by dissecting the seedlings in each pot and counting the number of live and dead larvae from representative plants at 8 DAH. Plants were allowed to grow to record the yield data. As a negative control, plants were treated with water, similar to the CA-treated plants, for data collection.

### Statistics and reproducibility

Larval and pupal length measurements, leaf length measurements, and yield data are represented as mean ± s.e.m with data collected from *n* = 6 to 12 plants. Metabolite and polyamine profiling was done with three biological replicates with at least 10 plants per replicate. NR staining was done with at least *n* = 5 plants per genotype per time point. Statistical analysis (unpaired two-tailed t-test) was performed with GraphPad Prism (v10). Gene expression data are presented as natural log (ln) fold change ± s.e. for three biological replicates and three averaged technical replicates per biological replicate. Significant differences in means were determined by ANOVA using the PROC MIXED procedure of SAS^63^. No differences were considered statistically significant when *p-*values were greater than 0.05.

### Reporting summary

The reporting summary is provided in the Nature Reporting Summary linked to this article. Further information on research design is available in the Nature Portfolio Reporting Summary linked to this article.

## Supplementary information

Supplementary Information

Description of Additional Supplementary Files

Supplementary Data 1

Supplementary Data 2

Supplementary Data 3

Supplementary Data 4

Supplementary Data 5

Supplementary Data 6

Supplementary Data 7

Reporting Summary

## Data Availability

All data are provided within the manuscript and the Supplementary Information files. Supplementary Data 1 contains the processed Metabolomics Data, and Supplementary Data 2 provides the qRT-PCR primers for gene expression studies. Source data associated with Figs. 1–4 and 6 are provided in Supplementary Data 3–6. Source data related to Supplementary Figs. 1 and 3 are provided in Supplementary Data 7. The raw metabolomics data is available at the NIH common Fund’s National Metabolomics Data Repository (NMDR) website, the Metabolomics Workbench, https://metabolomicsworkbench.org/ where it has been assigned Project ID (PR002946). The data can be accessed directly via it’s Project https://doi.org/10.21228/M8VV9P. All data are available from the corresponding author on reasonable request.

## References

[1] Pauly, P. J. Fighting the Hessian fly: American and British responses to insect invasions, 1776-1789. *Environ. His.* **7**, 486–506 (2002).

[2] Schmid, R. B., Knutson, A., Giles, K. L. & McCornack, B. P. Hessian fly (Diptera: Cecidomyiidae) biology and management in wheat. *J. Integr. Pest Manag.* **9**, 14 (2018).

[3] Tedesse, W. et al. Wheat breeding for Hessian fly resistance at ICARDA. *Crop J.* **10**, 1528–1535 (2022).

[4] Wang, Z. et al. An insect nucleoside disphosphate kinase (NDK) functions as an effector protein in wheat-Hessian fly interactions. *Insect Biochem. Mol. Biol.* **100**, 30–38 (2018). 10.1016/j.ibmb.2018.06.003 29913225

[5] Subramanyam, S., Nemacheck, J. A., Bernal-Crespo, V. & Sardesai, N. Insect derived extra oral GH32 plays a role in susceptibility of wheat to Hessian fly. *Sci. Rep.* **11**, 2081 (2021). 10.1038/s41598-021-81481-4 PMC7822839 33483565

[6] Hatchett, J. H. & Gallun, R. L. Genetics of the ability of the Hessian fly, *Mayetiola destructor*, to survive on wheats having different genes for resistance. *Ann. Entomol. Soc. Am.* **63**, 1400–1407 (1970).

[7] Stuart, J. J., Chen, M.-S., Shukle, R. & Harris, M. O. Gall midges (Hessian flies) as plant pathogens. *Annu. Rev. Phytopathol.* **50**, 339–357 (2012). 10.1146/annurev-phyto-072910-095255 22656645

[8] Zhao, C. et al. A massive expansion of effector genes underlies gall-formation in the wheat pest *Mayetiola destructor*. *Curr. Biol.* **25**, 613–620 (2015). 10.1016/j.cub.2014.12.057 25660540

[9] Ratcliffe, R. H. et al. Biotype composition of Hessian fly (Diptera: Cecidomyiidae) populations from the southeastern, midwestern, and northwestern United States and virulence to resistance genes in wheat. *J. Econ. Entomol.* **93**, 1319–1328 (2000). 10.1603/0022-0493-93.4.1319 10985049

[10] Zhao, L. et al. Identification of two novel Hessian fly resistance genes H35 and H36 in a hard winter wheat line SD06165. *Theor. Appl. Genet.* **133**, 2343–2353 (2020). 10.1007/s00122-020-03602-3 32436021

[11] Cambron, S. E. et al. Virulence in Hessian fly (Diptera: Cecidomyiidae) field collections from the southeastern United States to 21 resistance genes in wheat. *J. Econ. Entomol.* **103**, 2229–2235 (2010). 10.1603/ec10219 21309248

[12] Schemerhorn, B. J., Cambron, S. E. & Subramanyam, S. Hessian fly (Dipetera: Cecidomyiidae) virulence in Louisiana assessment of field populations from 2023 to efficacy of 27 Hessian fly resistance genes in wheat. *J. Econ. Entomol.* **117**, 660–665 (2024). 10.1093/jee/toae002 38254238

[13] Rosenthal, J. P. & Kotanen, P. M. Terrestrial plant tolerance to herbivory. *Trends Ecol. Evol.* **9**, 145–148 (1994). 10.1016/0169-5347(94)90180-5 21236799

[14] Peterson, R. K. D., Varella, A. C. & Higley, L. G. Tolerance: the forgotten child of plant resistance. *PeerJ* **5**, e3934 (2017). 10.7717/peerj.3934 PMC5647859 29062607

[15] Painter, R. H. *Insect Resistance in Crop Plants* (Macmillan, 1951).

[16] Painter, R. H., Jones, E. T., Johston, C. O. & Parker, J. H. Transference of Hessian fly resistance and other characteristics of Marquillo spring wheat to winter wheat. *Tech. Bull. Kans. Agric. Exp. Stn.* **49**, 3–55 (1940).

[17] Reese, J. C., Schwenke, J. R., Lamont, P. S. & Zehr, D. D. Importance and quantification of plant tolerance in crop management programs for aphids: greenbug resistance in sorghum. *J. Agric. Entomol.* **11**, 255–270 (1994).

[18] Winn, Z. J. et al. Mapping of a novel major effect Hessian fly field partial-resistance locus in southern soft red winter wheat line LA03136E7. *Theor. Appl. Genet.* **134**, 3911–3923 (2021). 10.1007/s00122-021-03936-6 34374831

[19] Hao, Y. et al. Characterization of new loci for Hessian fly resistance in common wheat. *Theor. Appl. Genet.* **126**, 1067–1076 (2013). 10.1007/s00122-012-2037-x 23296492

[20] Maas, F. B. I. I. I., Patterson, F. L., Foster, J. E. & Hatchett, J. H. Expression and inheritance of resistance of ‘Marquillo’ wheat to Hessian fly biotype D. *Crop Sci.* **27**, 49–52 (1987).

[21] Dabba, S. D. et al. Registration of durum wheat (*Triticum turgidum* ssp. *durum*) source of resistance to Hessian fly. *J. Plant Regist.* **14**, 445–449 (2020).

[22] Subramanyam, S. & Nemacheck, J. N. New sources of Hessian fly resistance in *Triticum turgidum* wheat lines from Asia and Europe. *Genet. Resour. Crop Evol.* **70**, 1341–1347 (2023).

[23] Subramanyam, S. et al. Expression of two wheat defense-response genes, Hfr-1 and Wci-1, under biotic and abiotic stresses. *Plant Sci.* **170**, 90–103 (2006).

[24] Giovanini, M. P. et al. A novel wheat gene encoding a putative chitin-binding lectin is associated with resistance against Hessian fly. *Mol. Plant Pathol.* **8**, 69–82 (2007). 10.1111/j.1364-3703.2006.00371.x 20507479

[25] Subramanyam, S., Zheng, C., Shukle, J. T. & Williams, C. E. Hessian fly larval attack triggers elevated expression of disease resistance dirigent-like protein-encoding gene, *HfrDrd* in resistant wheat. *Arthropod Plant Interact.* **7**, 389–402 (2013).

[26] Liu, X. et al. Wheat *Mds-1* encodes a heat-shock protein and governs susceptibility towards the Hessian fly gall midge. *Nat. Commun.* **4**, 2070 (2013). 10.1038/ncomms3070 23792912

[27] Subramanyam, S. et al. Hessian fly larval feeding triggers enhanced polyamine levels in susceptible but not resistant wheat. *BMC Plant Biol.* **15**, 3 (2015). 10.1186/s12870-014-0396-y PMC4308891 25592131

[28] Williams, C. W. et al. Induced epidermal permeability modulates resistance and susceptibility of wheat seedlings to herbivory by Hessian fly larvae. *J. Exp. Bot.* **62**, 4521–4531 (2011). 10.1093/jxb/err160 PMC3170548 21659664

[29] Kiszonas, A. M. et al. Agronomic traits in durum wheat germplasm possessing puroindoline genes. *Agron. J.* **111**, 1254–1265 (2019).

[30] Morris, C. F. et al. Soft kernel durum wheat—a new bakery ingredient? *Cereal Foods World* **60**, 76–83 (2015).

[31] Vendramin, V. et al. Genomic tools for durum wheat breeding: de novo assembly of Svevo transcriptome and SNP discovery in elite germplasm. *BMC Genom.* **20**, 278 (2019). 10.1186/s12864-019-5645-x PMC6456968 30971220

[32] DeSantis, M. A., Soccio, M., Laus, M. N. & Flagella, Z. Influence of drought and salt stress on durum wheat grain quality and composition: a review. *Plants* **10**, 2599 (2021). 10.3390/plants10122599 PMC8708103 34961071

[33] Zhou, X. et al. Genome-wide mapping of loci for adult-plant resistance to stripe rust in durum wheat Svevo using the 90k SNP array. *Plant Dis.* **105**, 879–888 (2021). 10.1094/PDIS-09-20-1933-RE 33141640

[34] Zhou, X. et al. Fine mapping of QYrsv.swust-1BL for resistance to stripe rust in durum wheat Svevo. *Front. Plant Sci.* **15**, 1395223 (2024). 10.3389/fpls.2024.1395223 PMC11204296 38933466

[35] Garcia, L. C. & Eubanks, M. D. Overcompensation for insect herbivory: a review and meta-analysis of the evidence. *Ecology* **100**, e02585 (2019). 10.1002/ecy.2585 30554427

[36] Chen, M.-S. et al. A group of related cDNAs encoding secreted proteins from Hessian fly [*Mayetiola destructor* (Say)] salivary glands. *Insect Mol. Biol.* **13**, 101–108 (2004). 10.1111/j.1365-2583.2004.00465.x 14728671

[37] Subramanyam, S. et al. Functional characterization of HFR1, a high-mannose N-glycan-specific wheat lectin induced by Hessian fly larvae. *Plant Physiol.* **147**, 1412–1426 (2008). 10.1104/pp.108.116145 PMC2442546 18467454

[38] Sardesai, N., Subramanyam, S., Nemacheck, J. A. & Williams, C. E. Modulation of defense-response gene expression in wheat during Hessian fly larval feeding. *J. Plant Interact.* **1**, 39–50 (2005).

[39] Liu, X. et al. Reactive oxygen species are involved in plant defense against a gall midge. *Plant Physiol.* **152**, 985–999 (2010). 10.1104/pp.109.150656 PMC2815885 19965963

[40] Zhu, L. et al. Hessian fly (*Mayetiola destructor*) attack causes a dramatic shift in carbon and nitrogen metabolism in wheat. *Mol. Plant Microbe Interact.* **21**, 70–78 (2008). 10.1094/MPMI-21-1-0070 18052884

[41] Harris, M. O. et al. Virulent Hessian fly (Diptera: Cecidomyiidae) larvae induce a nutritive tissue during compatible interactions with wheat. *Ann. Entomol. Soc. Am.* **99**, 305–316 (2006).

[42] Saltzmann, K. D., Giovanini, M. P., Zheng, C. & Williams, C. E. Virulent Hessian fly larvae manipulate the free amino acid content of host wheat plants. *J. Chem. Ecol.* **34**, 1401–1410 (2008). 10.1007/s10886-008-9544-x 18841417

[43] Subramanyam, S. et al. Modulation of nonessential amino acid biosynthetic pathways in virulent Hessian fly larvae (*Mayetiola destructor*), feeding on susceptible host wheat (*Triticum aestivum*). *J. Insect Physiol.* **105**, 54–63 (2018). 10.1016/j.jinsphys.2018.01.001 29336997

[44] Reshi, Z. F., Ahmed, W., Lukatkin, A. S. & Javed, S. B. From nature to lab: a review of secondary metabolite biosynthetic pathways, environmental influences, and in vitro approaches. *Metabolites* **13**, 895 (2023). 10.3390/metabo13080895 PMC10456650 37623839

[45] Jamieson, M. A. et al. Global change effects on plant-insect interactions: the role of phytochemistry. *Curr. Opin. Insect Sci.* **23**, 70–80 (2017). 10.1016/j.cois.2017.07.009 29129286

[46] Stasolla, C., Katahira, R., Thorpe, T. A. & Ashihara, H. Purine and pyrimidine nucleotide metabolism in higher plants. *J. Plant Physiol.* **160**, 1271–1295 (2003). 10.1078/0176-1617-01169 14658380

[47] Ren, X. et al. Gut symbiotic bacteria are involved in nitrogen recycling in the tephritid fruit fly *Bactrocera dorsalis*. *BMC Biol.* **20**, 201 (2022). 10.1186/s12915-022-01399-9 PMC9476588 36104720

[48] Bansal, R. et al. Hessian fly-associated bacteria: transmission, essentiality and composition. *PLoS ONE* **6**, e23170 (2011). 10.1371/journal.pone.0023170 PMC3156707 21858016

[49] Aranega-Bou, P. et al. Priming of plant resistance by natural compounds. Hexanoic acid as a model. *Front. Plant Sci.* **5**, 488 (2014). 10.3389/fpls.2014.00488 PMC4181288 25324848

[50] Bhardwaj, R. D., Kaur, L. & Srivastava, P. Comparative evaluation of different phenolic acids as priming agents for mitigating drought stress in wheat seedlings. *Proc. Natl. Acad. Sci. USA* **87**, 1133–1142 (2015).

[51] Chaudhary, D. et al. Priming—a state of alert against environmental stress challenges in plants. *Plant Gene* **43**, 100513 (2025).

[52] Mughal, A. et al. Exploring the role of caffeic acid in mitigating abiotic stresses in plants: a review. *Plant Stress* **12**, 100487 (2024).

[53] Návarová, H., Bernsdorff, F., Döring, A.-C. & Zeier, J. Pipecolic acid, an endogenous mediator of defense amplification and priming, is a critical regulator of inducible plant immunity. *Plant Cell* **24**, 5123–5141 (2012). 10.1105/tpc.112.103564 PMC3556979 23221596

[54] Chen, Y.-C. et al. N-hydroxy-pipecolic acid is a mobile metabolite that induces systemic disease resistance in *Arabidopsis*. *Proc. Natl. Acad. Sci. USA* **115**, E4920–E4929 (2018). 10.1073/pnas.1805291115 PMC6003486 29735713

[55] Wang, P. et al. A novel role of pipecolic acid biosynthetic pathway in drought tolerance through the antioxidant system in tomato. *Antioxidants* **10**, 1923 (2021). 10.3390/antiox10121923 PMC8750784 34943026

[56] Sun, L. et al. Role of serotonin in plant stress responses: Quo vadis? *J. Integr. Plant Biol.* **67**, 1706–1724 (2025). 10.1111/jipb.13882 40098453

[57] Poulton, J. E. Transmethylation and demethylation reactions in the metabolism of secondary plant products. in (ed. Conn, E. E.) *The Biochemistry of Plants* (Academic Press, 1981).

[58] Misra, V. Epigenetics for combating pests in plants: updated methods and current achievements. in (ed. Chen, J.-T.) *Epigenetics for Climate-Smart and Sustainable Agriculture* 536–552 (CAB International, 2025).

[59] Singh, R. P. et al. Challenges to wheat disease resistance and current global strategies. *Annu. Rev. Phytopathol.* **63**, 201–224 (2025). 10.1146/annurev-phyto-121923-082727 40388806

[60] Sosa, O. & Gallun, R. L. Purification of races B and C of the Hessian fly by genetic manipulation. *Ann. Entomol. Soc. Am.* **66**, 1065–1070 (1973).

[61] Subramanyam, S., Nemacheck, J. A., Suetsugu, T. E., Flynn, R. D. & Faik, A. Genetic and phenotypic responses of temperature-independent Hessian fly-resistant durum wheat to larval attack during heat stress. *BMC Plant Biol.* **25**, 210 (2025). 10.1186/s12870-025-06226-1 PMC11831824 39962422

[62] Nemacheck, J. A., Flynn, R. D., Zapf, K. & Subramanyam, S. Identification of new sources of Hessian fly resistance in tetraploid wheat. *Crop Sci.* **63**, 1354–1363 (2023).

[63] Hargarten, A. M. et al. Physical and metabolic consequences of Hessian fly infestation are more severe on nonhost *Brachypodium distachyon* than on host-plant resistant wheat. *Arthropod Plant Interact.* **11**, 767–783 (2017).

[64] Minocha, R. & Long, S. Simultaneous separation and quantitation of amino acids and polyamines of forest tree tissues and cell cultures within a single high-performance liquid chromatography run using dansyl derivatization. *J. Chromatogr. A* **1035**, 63–73 (2004). 10.1016/j.chroma.2004.02.026 15117075

[65] Grace, S. C. & Hudson, D. A. Processing and visualization of metabolomics data using R. in *Metabolomics—Fundamentals and Applications* (IntechOpen, 2016).

[66] Bender, R. & Lange, S. Adjusting for multiple testing—when and how? *J. Clin. Epidemiol.* **54**, 343–349 (2001). 10.1016/s0895-4356(00)00314-0 11297884

[67] Xi, B., Gu, H., Beniasadi, H. & Raftery, D. Statistical analysis and modeling of mass spectrometry based metabolomics data. *Methods Mol. Biol.* **1198**, 333–353 (2014). 10.1007/978-1-4939-1258-2_22 PMC4319703 25270940

[68] Antunes, A. C. et al. Untargeted metabolomics of strawberry (Fragaria x ananassa ‘Camarosa’) fruit from plants grown under osmotic stress conditions. *J. Sci. Food Agric.* **99**, 6973–6980 (2019). 10.1002/jsfa.9986 31414485

[69] Wang, K.-D., Borrego, E. J., Kenerley, C. M. & Kolomiets, M. V. Oxylipins other than jasmonic acid are xylem-resident signals regulating systemic resistance induced by trichoderma virens in maize. *Plant Cell* **32**, 166–185 (2020). 10.1105/tpc.19.00487 PMC6961617 31690653

