# Supplementary material for: Hessian fly tolerance increases yield in elite durum wheat cultivars Svevo and Soft Svevo
Source: Commun Biol. 2026 Jun 15;9:1277. doi: 10.1038/s42003-026-10494-4 (PMC13631361; doi:10.1038/s42003-026-10494-4)
Supplement: Supplementary file 2 [file 42003_2026_10494_MOESM1_ESM.pdf]

Hessian fly tolerance increases yield in elite durum wheat cultivars Svevo and Soft Svevo

Rachel D. Flynn[1,2], Devender Arora[3], Kumar Shrestha[4], Joe Louis[4,5], Subhash C. Minocha[6], Steven S. Xu[7], Subhashree Subramanyam [1,2]✉

✉ Corresponding author
Subhashree Subramanyam,
Email: Subhashree.Subramanyam@usda.gov

This Supplementary Information PDF file includes:

Supplementary Figures 1 – 8
Supplementary Methods

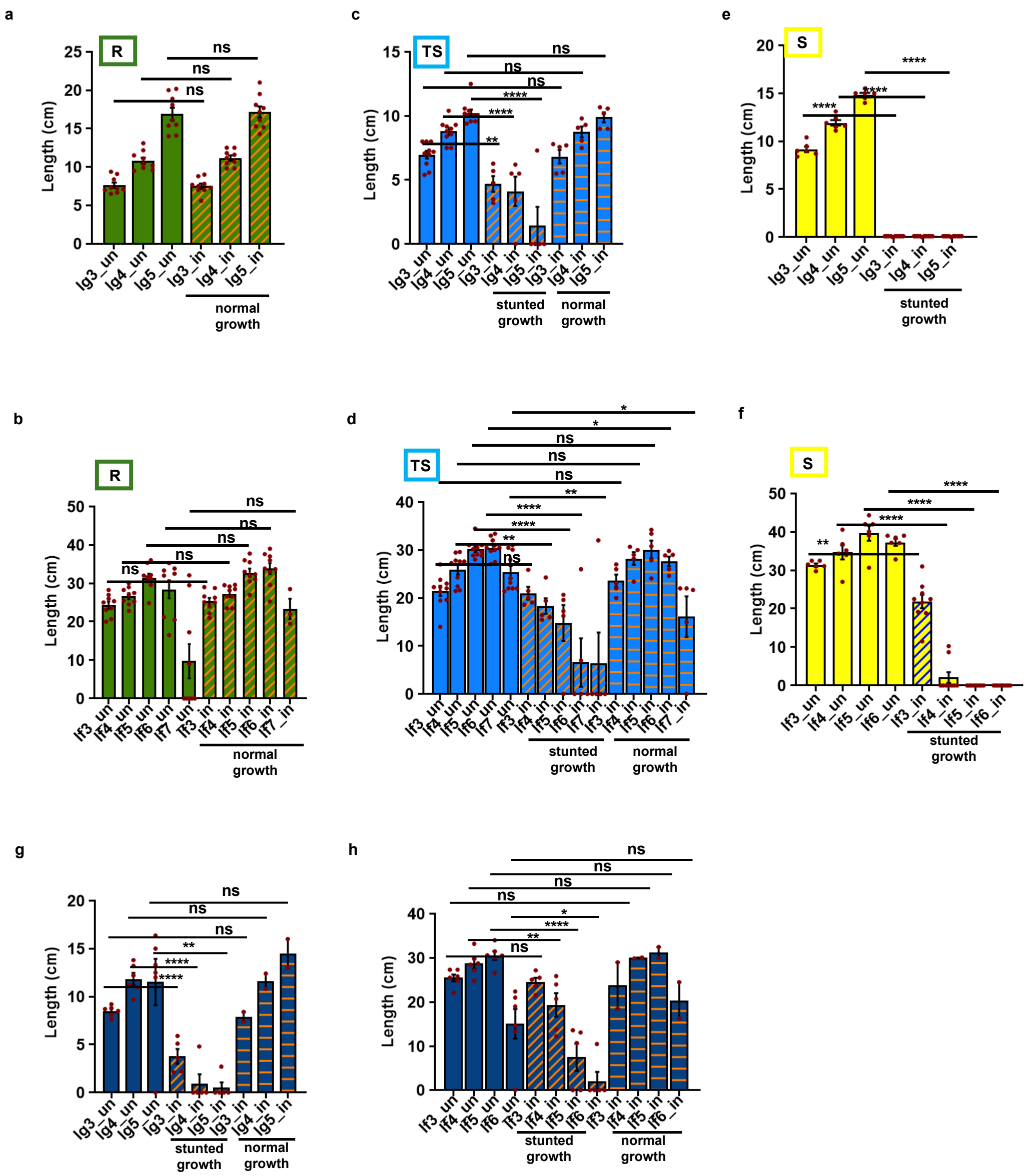

Supplementary Fig. 1 Ligule and leaf growth in Hessian fly infested wheat. Length of different ligule (lg) and leaf (lf) from uninfested (un) and biotype L-infested (in) PI 519832 (resistant, R), Svevo (tolerant, TS), Soft Svevo (tolerant, TSS), and PI 61188 (susceptible, S) wheat genotypes at 22 DAH (days after egg hatch). **a** ligule length in R plants. **b** leaf length in R plants. **c** ligule length in TS plants. **d** leaf length in R plants. **e** ligule length in S plants. **f** leaf length in S plants. **g** ligule length in TSS plants. **h** leaf length in TSS plants. Statistical analysis (unpaired two-tailed *t* test) was performed with GraphPad Prism (v10). Lines above the bars represent significant comparisons between R, TS, S and TSS: black lines indicate comparisons for Un (uninfested) vs In (infested) within a genotype. The number (n) of replicates for each experiment are provided in Supplementary Data 7. Error bar represents the standard error of mean. $*p \leq 0.05$, $**p \leq 0.01$, $***p \leq 0.001$, and $****p \leq 0.0001$.

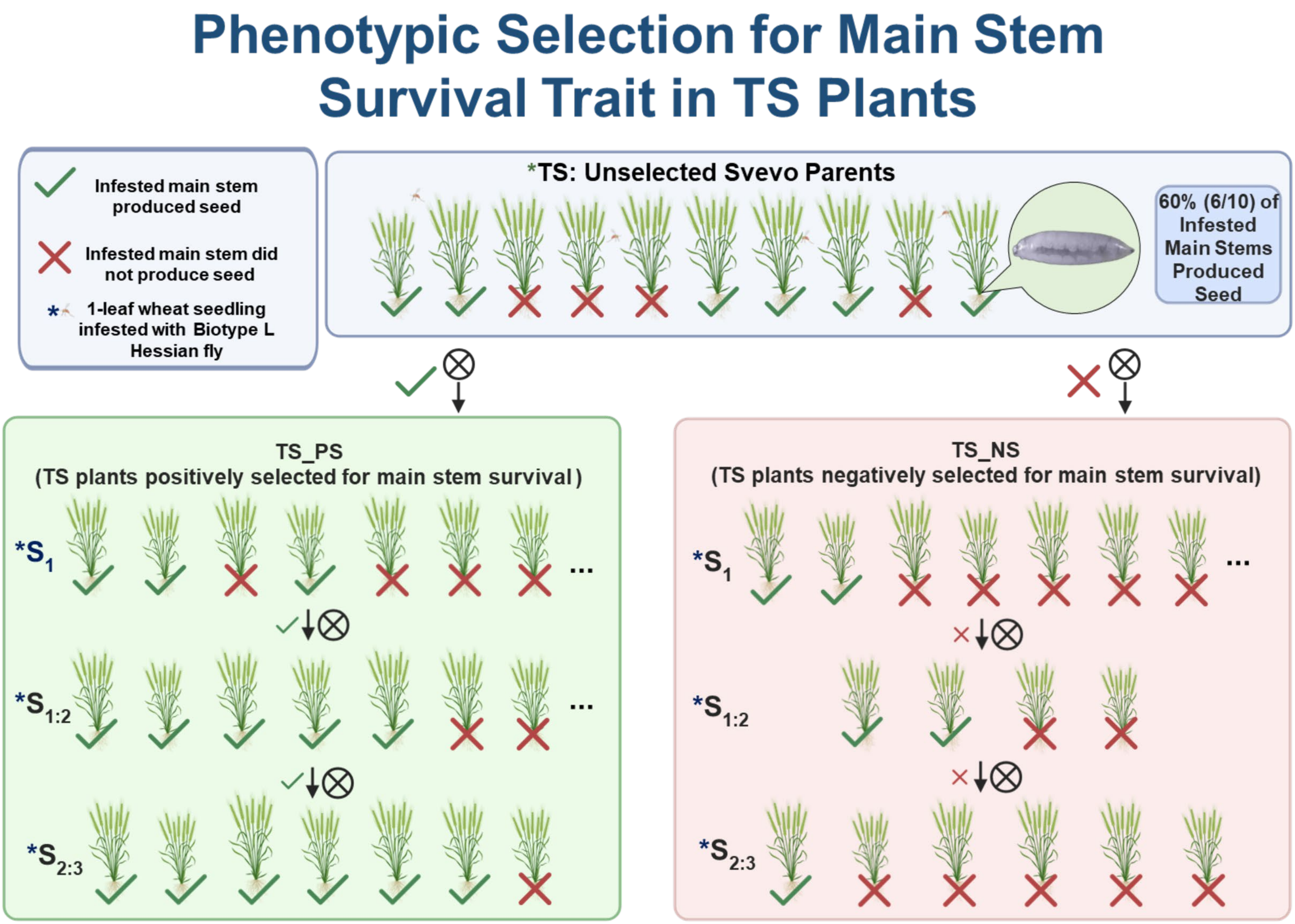

Supplementary Fig. 2. **Main stem survival trait selection in Svevo wheat.** Schematic representation showing overview of phenotypic selection for increasing or decreasing main stem (MS, the primary biotype L-infested stem) survival trait in Svevo (tolerant, TS) durum wheat genotype. Seed production by MS post-infestation was used as a phenotypic marker for selection. Green '✓' indicate plants with MS producing seeds after infestation, while the red 'X' depict plants with MS that did not produce seed after infestation. Green background illustrates positive selection in TS plants (TS_PS) and the red background indicates negative selection in TS plants (TS_NS) for MS survival trait. The circle and x symbol next to the black arrow denotes self-pollination. *$S_{\#:\#}$ indicates an infested, self-crossed generation originating from an individually selected plant, with the first number being the source generation and the second being the represented generation. The number of plants along with percentage of plants showing the selected trait are shown for each generation. The schematic diagram was prepared using BioRender Scientific Image and Illustration Software (BioRender citation: https://BioRender.com/zzozg8z).

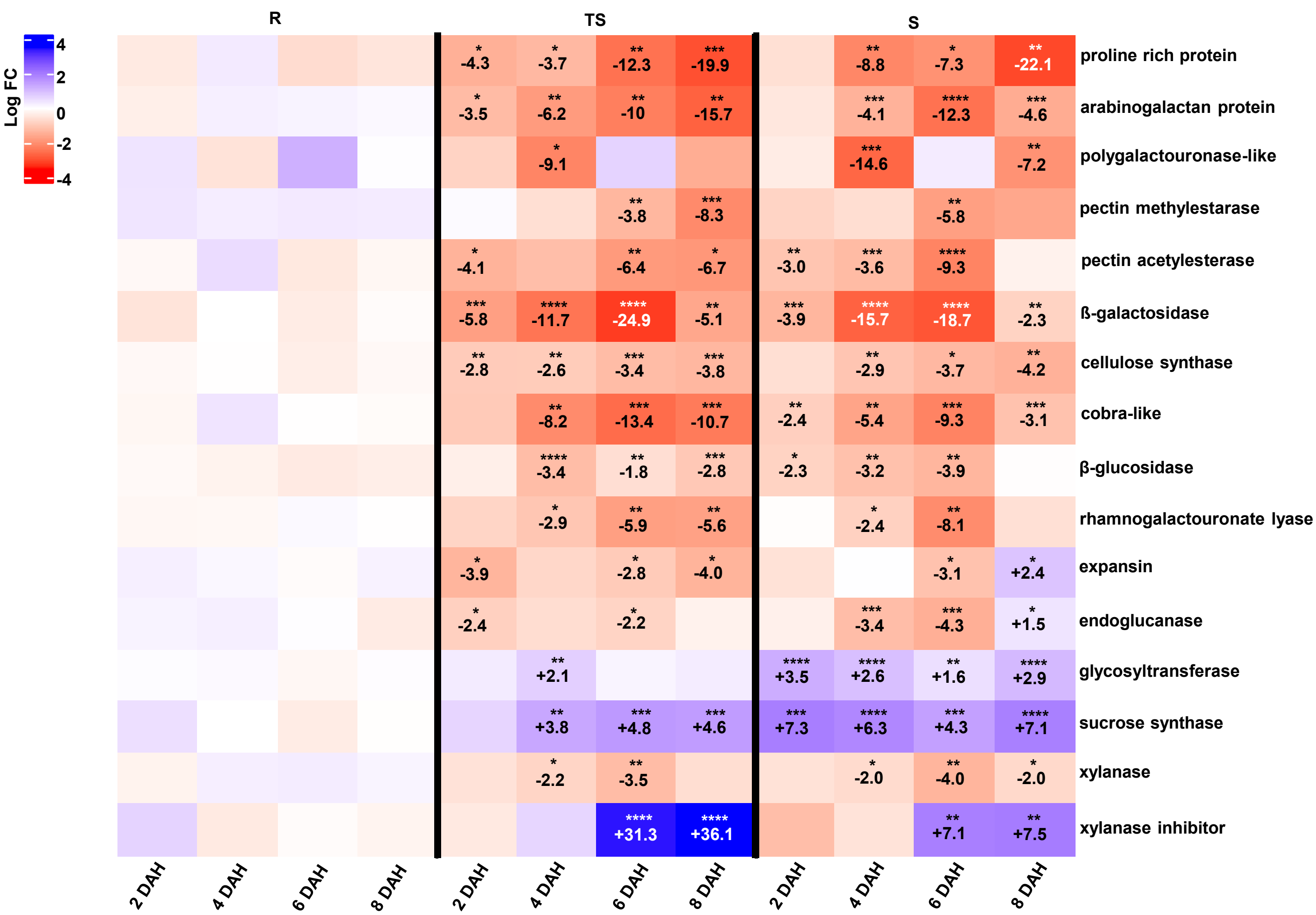

Supplementary Fig. 3. Temporal expression of cell wall genes in durum wheat to Hessian fly larval feeding. Heatmap depicts the expression profile of 16 genes encoding for cell wall-associated proteins in biotype L-infested PI 519832 (resistant, R), Svevo (TS, tolerant), and PI 61188 (S, susceptible) durum wheat genotypes at 2, 4, 6, and 8 DAH (days after egg hatch) time points. Data is shown as logarithmic fold change (log_eFC) of infested vs uninfested control for each genotype and each time point. Data represent three biological replicates and three averaged technical replicates per biological replicate and are provided in Supplementary Data 7. Significant differences are indicated by asterisks as determined by ANOVA using the PROC MIXED procedure of SAS[63]. Only the significant fold changes ($p \leq 0.05$, fold change ≥ 2, are indicated as numbers within the cells in the heatmap, *$p \leq 0.05$, **$p \leq 0.01$, ***$p \leq 0.001$, and ****$p \leq 0.0001$. Blue (+) indicates upregulated genes and red (-) indicates downregulated genes in the heatmap.

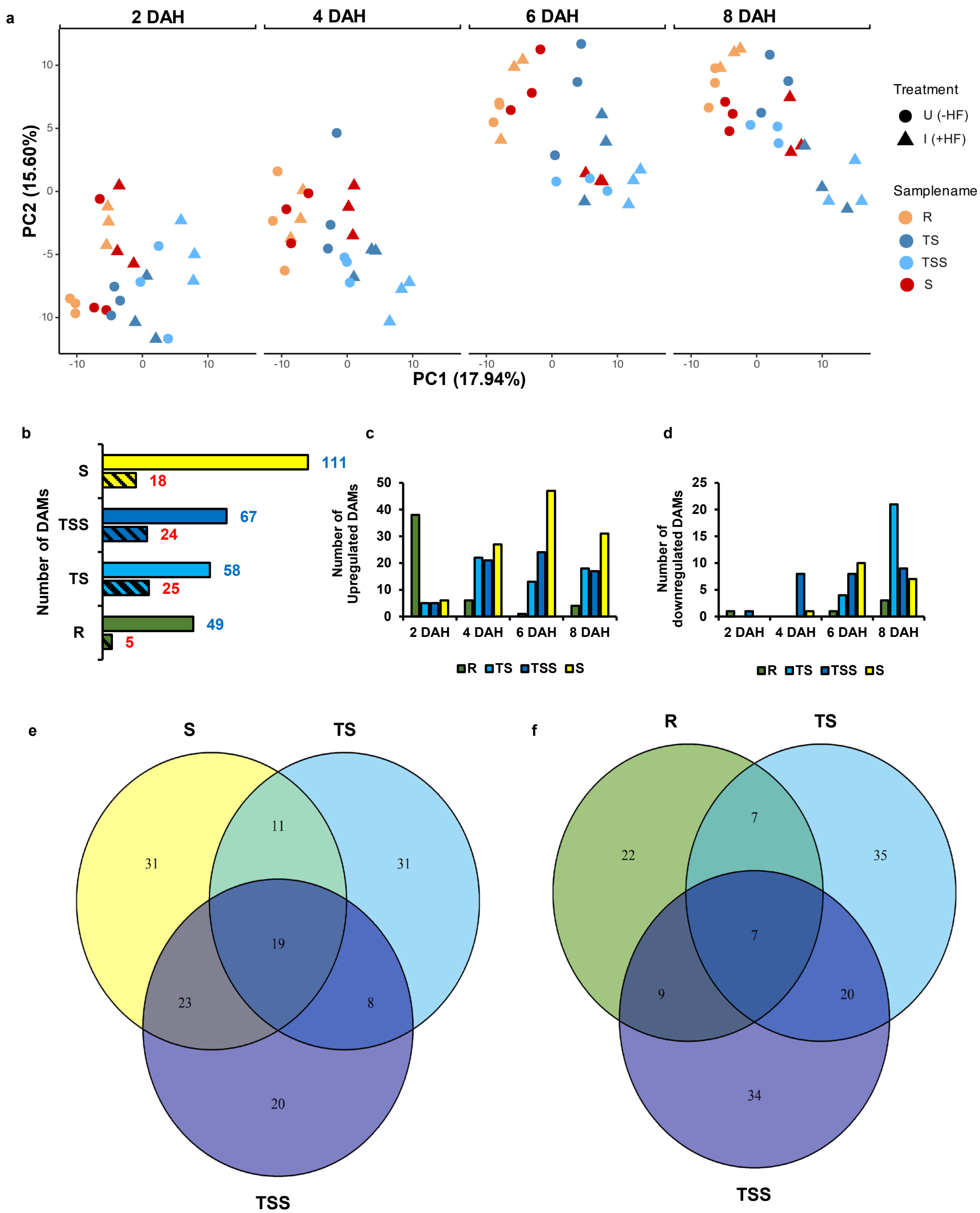

Supplementary Fig. 4. Differential accumulation of metabolites in durum wheat to Hessian fly larval feeding. a Principal component analysis of all 410 metabolites accumulated in the uninfested (U -HF, ●) and biotype L-infested (I +HF, ▲) PI 519832 (R, resistant, orange); Svevo (TS, tolerant, dark blue); Soft Svevo (TSS, tolerant, light blue) and PI 61188 (S, susceptible, red) wheat genotypes and grouped by time points (2, 4, 6, and 8) DAH (days after egg hatch). **b** Total number of differentially accumulated metabolites (DAMs) in R (green), TS (aqua), TSS (dark blue) and S (yellow) wheat genotypes. Solid bars indicate upregulated and diagonal stripes bar indicate downregulated DAMs. **c-d** Number of upregulated (left graph) and downregulated (right graph) DAMs in R (green), TS (aqua), TSS (dark blue) and S (yellow) wheat genotypes. **e** Venn diagram of upregulated DAMs depicting shared and uniquely accumulated number of metabolites between TS (light blue), TSS (dark blue) and S (yellow) wheat genotypes. **f** Venn diagram of downregulated DAMs depicting shared and uniquely accumulated number of metabolites between TS (light blue), TSS (dark blue) and R (green) wheat genotypes. Data shown are from three biological replicates. DAMs considered statistically significant having $\log_2$FC (foldchange) ≥ 2 and $p \leq 0.05$ in infested vs uninfested controls.

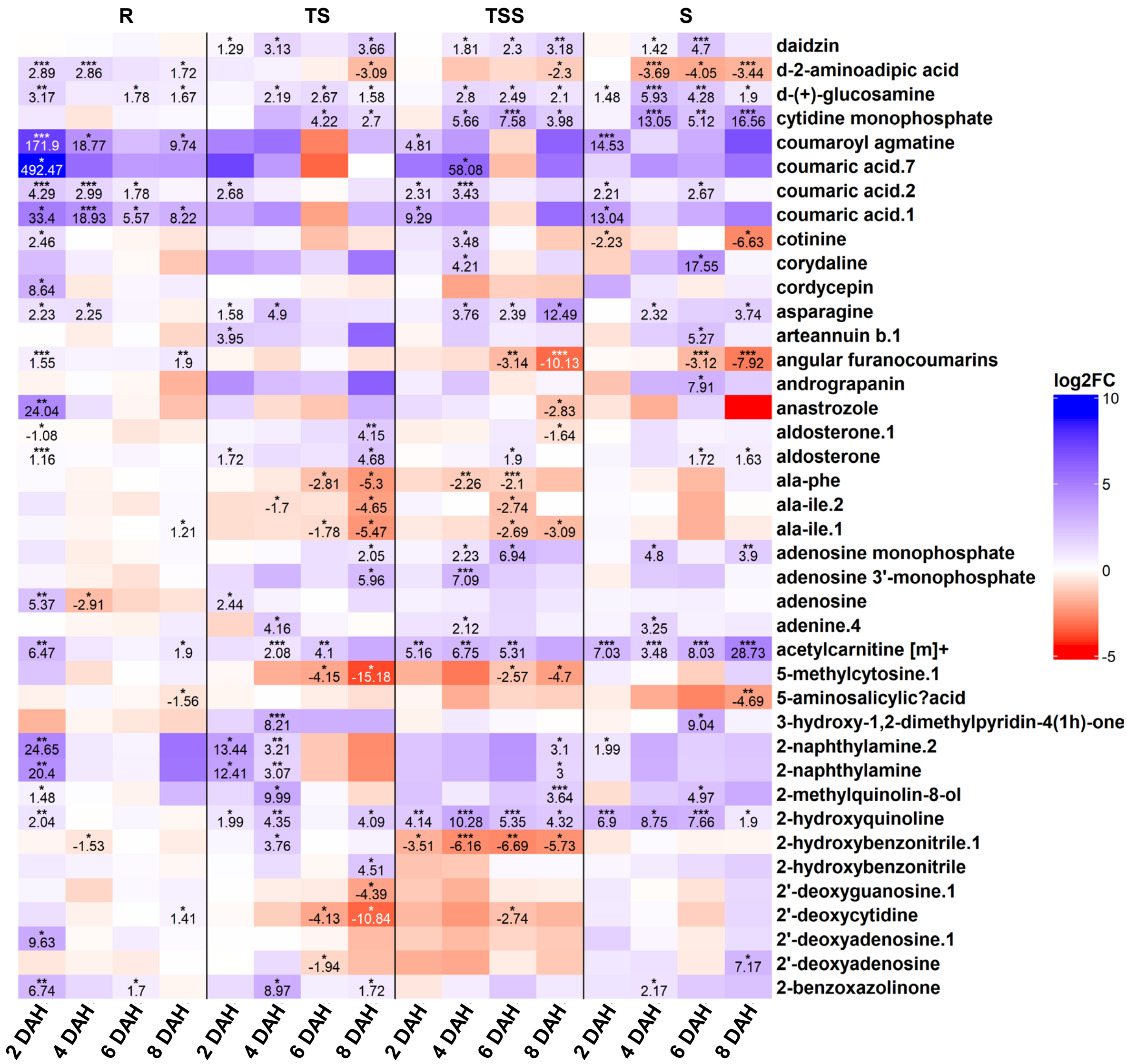

Supplementary Fig. 5. Differential accumulation of metabolites in durum wheat to Hessian fly larval feeding. Heatmap showing 40 differentially accumulated metabolites (DAMs) in biotype L-infested PI 519832 (R, resistant); Svevo (TS, tolerant); Soft Svevo (TSS,) and PI 61188 (S, susceptible) wheat genotypes at 2, 4, 6, and 8 DAH (days after egg hatch) time points. Data from three biological replicates are represented as logarithmic fold change (log_2FC) of infested vs uninfested control for each metabolite/per genotype/per time point. Only the significant (*$p \leq 0.05$, **$p \leq 0.01$, ***$p \leq 0.001$, and ****$p \leq 0.0001$) metabolites showing $log_2FC \geq 2$ (infested vs uninfested) are indicated as numbers within the heatmap. Blue indicates upregulated and red indicates downregulated DAMs.

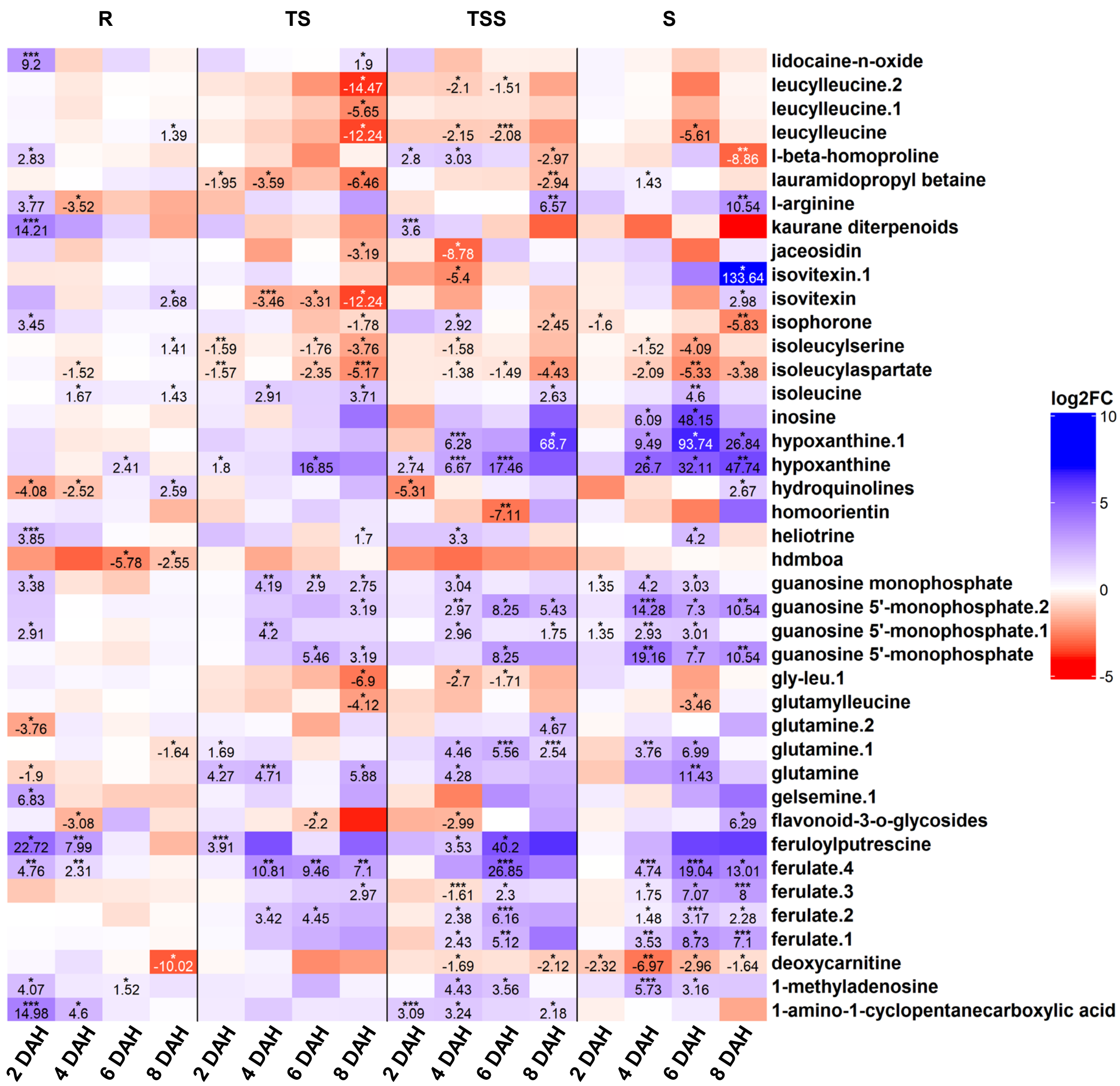

Supplementary Fig. 6. Differential accumulation of metabolites in durum wheat to Hessian fly larval feeding. Heatmap showing 41 differentially accumulated metabolites (DAMs) in biotype L-infested PI 519832 (R, resistant); Svevo (TS, tolerant); Soft Svevo (TSS,) and PI 61188 (S, susceptible) wheat genotypes at 2, 4, 6, and 8 DAH (days after egg hatch) time points. Data from three biological replicates are represented as logarithmic fold change (log_2FC) of infested vs uninfested control for each metabolite/per genotype/per time point. Only the significant (*$p \leq 0.05$, **$p \leq 0.01$, ***$p \leq 0.001$, and ****$p \leq 0.0001$) metabolites showing $log_2FC \geq 2$ (infested vs uninfested) are indicated as numbers within the heatmap. Blue indicates upregulated and red indicates downregulated DAMs.

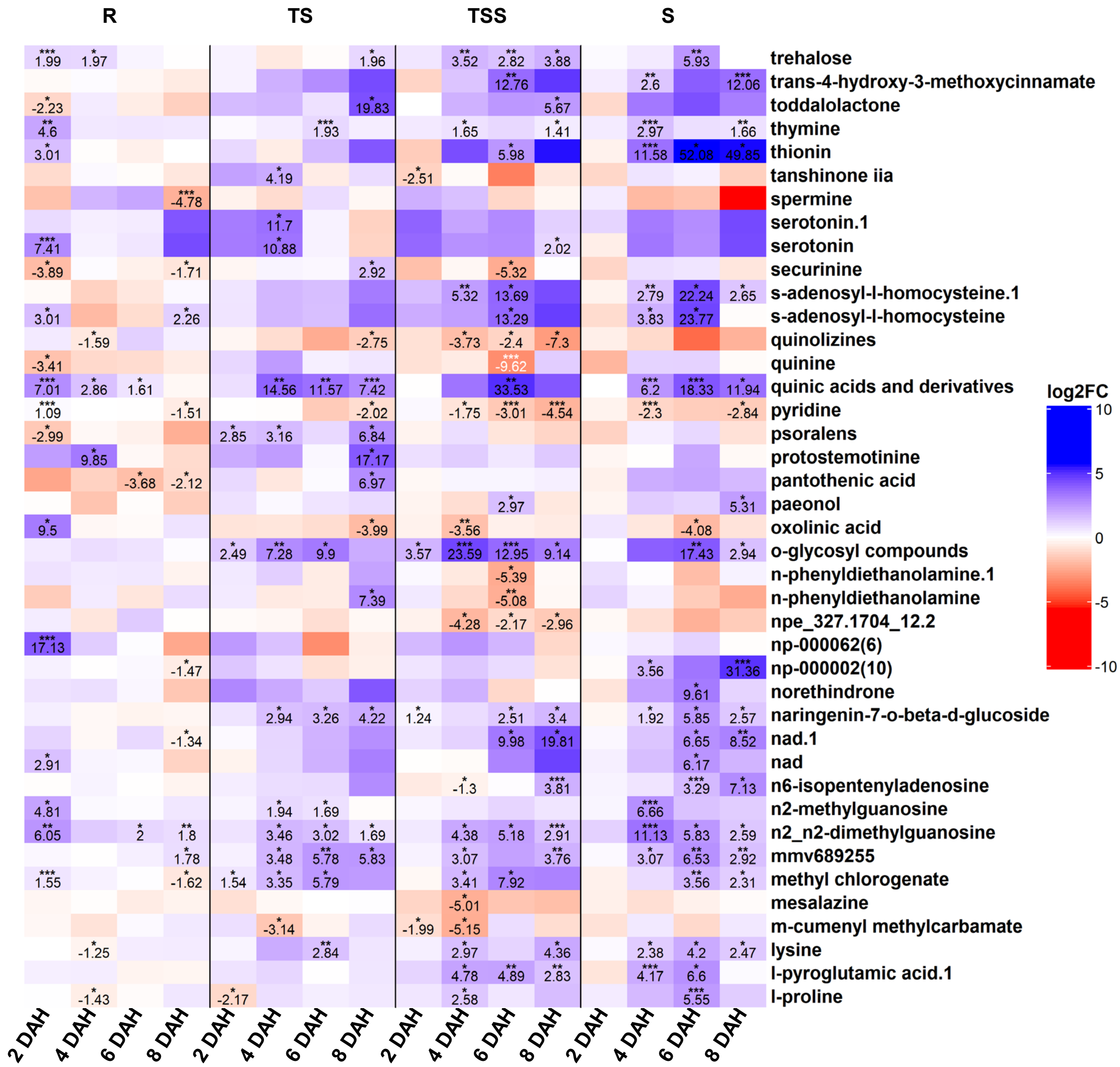

Supplementary Fig. 7. Differential accumulation of metabolites in durum wheat to Hessian fly larval feeding. Heatmap showing 41 differentially accumulated metabolites (DAMs) in biotype L-infested PI 519832 (R, resistant); Svevo (TS, tolerant); Soft Svevo (TSS,) and PI 61188 (S, susceptible) wheat genotypes at 2, 4, 6, and 8 DAH (days after egg hatch) time points. Data from three biological replicates are represented as logarithmic fold change (log_2FC) of infested vs uninfested control for each metabolite/per genotype/per time point. Only the significant (*$p \leq 0.05$, **$p \leq 0.01$, ***$p \leq 0.001$, and ****$p \leq 0.0001$) metabolites showing $log_2FC \geq 2$ (infested vs uninfested) are indicated as numbers within the heatmap. Blue indicates upregulated and red indicates downregulated DAMs.

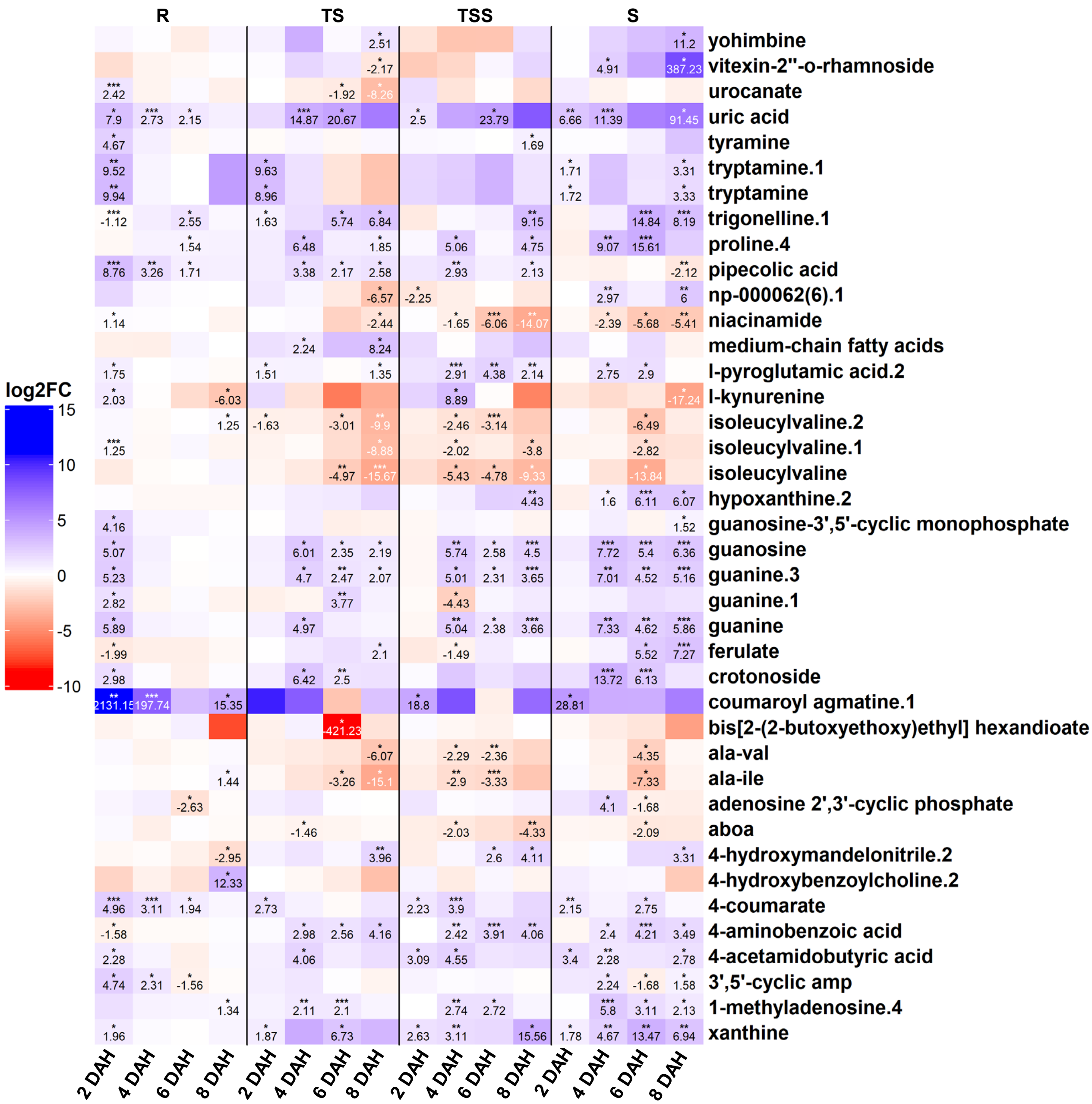

Supplementary Fig. 8. Differential accumulation of metabolites in durum wheat to Hessian fly larval feeding. Heatmap showing 40 differentially accumulated metabolites (DAMs) in biotype L-infested PI 519832 (R, resistant); Svevo (TS, tolerant); Soft Svevo (TSS,) and PI 61188 (S, susceptible) wheat genotypes at 2, 4, 6, and 8 DAH (days after egg hatch) time points. Data from three biological replicates are represented as logarithmic fold change (log_2FC) of infested vs uninfested control for each metabolite/per genotype/per time point. Only the significant (**p* ≤ 0.05, ***p* ≤ 0.01, ****p* ≤ 0.001, and *****p* ≤ 0.0001) metabolites showing $log_2FC \geq 2$ (infested vs uninfested) are indicated as numbers within the heatmap. Blue indicates upregulated and red indicates downregulated DAMs.

Supplementary Methods

Metabolomics Data Collection

MS-DIAL ver. 4.9.221218

#Project
MS1 Data type Profile
MS2 Data type Profile
Ion mode Positive
Target Metablomics
Mode ddMSMS

#Data collection parameters
Retention time begin 0.9
Retention time end 39
Mass range begin 0
Mass range end 2000
MS2 mass range begin 0
MS2 mass range end 2000

#Centroid parameters
MS1 tolerance 0.01
MS2 tolerance 0.025

#Isotope recognition
Maximum charged number 2

#Data processing
Number of threads 4

#Peak detection parameters
Smoothing method LinearWeightedMovingAverage
Smoothing level 3
Minimum peak width 5
Minimum peak height 500

#Peak spotting parameters
Mass slice width 0.1
Exclusion mass list (mass & tolerance)

#Deconvolution parameters
Sigma window value 0.5
MS2Dec amplitude cut off 0
Exclude after precursor True
Keep isotope until 0.5

Keep original precursor isotopes False

#MSP file and MS/MS identification setting
MSP file D:\Users\Workstation\Desktop\MSDIALLibraries (MSP)\MSMS_Public_EXP_Pos_VS17.msp
Retention time tolerance 100
Accurate mass tolerance (MS1) 0.005
Accurate mass tolerance (MS2) 0.01
Identification score cut off 80
Using retention time for scoring False
Using retention time for filtering False

#Text file and post identification (retention time and accurate mass based) setting
Text file
Retention time tolerance 0.3
Accurate mass tolerance 0.01
Identification score cut off 85

#Advanced setting for identification
Relative abundance cut off 0
Top candidate report False

#Adduct ion setting
[M+H]+
[M+Na]+
[M+2H]2+
[M+H+Na]2+

#Alignment parameters setting
Reference file Z:\Q-TOF\Data\Shubha\(47)_ST-4d-U-B.d
Retention time tolerance 0.3
MS1 tolerance 0.015
Retention time factor 0.5
MS1 factor 0.5
Peak count filter 0
N% detected in at least one group 50
Remove feature based on peak height fold-change True
Sample max / blank average 5
Sample average / blank average 5
Keep identified and annotated metabolites False
Keep removable features and assign the tag for checking True
Gap filling by compulsion True

#Tracking of isotope labels
Tracking of isotopic labels FALSE

#Ion mobility

Ion mobility data	FALSE
