# Supplementary material for: Hessian fly tolerance increases yield in elite durum wheat cultivars Svevo and Soft Svevo
Source: Commun Biol. 2026 Jun 15;9:1277. doi: 10.1038/s42003-026-10494-4 (PMC13631361; doi:10.1038/s42003-026-10494-4)
Supplement: Supplementary file 3 [file 42003_2026_10494_MOESM2_ESM.pdf]

Description of Additional Supplementary Files

File name: Supplementary Data 1

Description: Supplementary Table: Metabolomics source data

File name: Supplementary Data 2

Description: Supplementary Table: qRT-PCR primers used for gene expression studies

File name: Supplementary Data 3

Description: Source data behind Fig. 1 and Fig. 2

File name: Supplementary Data 4

Description: Source data behind Fig. 3

File name: Supplementary Data 5

Description: Source data behind Fig. 4

File name: Supplementary Data 6

Description: Source data behind Fig. 6

File name: Supplementary Data 7

Description: Source data behind Supplementary Fig.1 and Fig.3
